# Molecular Networking Revealed Unique UV-Absorbing Phospholipids: Favilipids from the Marine Sponge *Clathria faviformis*

**DOI:** 10.3390/md21020058

**Published:** 2023-01-18

**Authors:** Silvia Scarpato, Roberta Teta, Paola De Cicco, Francesca Borrelli, Joseph R. Pawlik, Valeria Costantino, Alfonso Mangoni

**Affiliations:** 1 Dipartimento di Farmacia, Università degli Studi di Napoli Federico II, Via Domenico Montesano 49, 80131 Napoli, Italy; 2 Dipartimento di Medicina Veterinaria e Produzioni Animali, Università degli Studi di Napoli Federico II, Via F. Delpino, 80137 Napoli, Italy; 3 Department of Biology and Marine Biology and Center for Marine Science, University of North Carolina Wilmington, 5600 Marvin K Moss Lane, Wilmington, NC 28409, USA

**Keywords:** natural products, marine sponges, sunscreen, secondary metabolites, LC-MS, molecular networking, phospholipids, UV-protecting properties, kinase inhibitor, *Clathria faviformis*

## Abstract

Analysis of extracts of the marine sponge *Clathria faviformis* by high-resolution LC-MS^2^ and molecular networking resulted in the discovery of a new family of potentially UV-protecting phospholipids, the favilipids. One of them, favilipid A (**1**), was isolated and its structure determined by mass and tandem mass spectrometry, NMR, electronic circular dichroism (ECD), and computational studies. Favilipid A, which has no close analogues among natural products, possesses an unprecedented structure characterized by a 4-aminodihydropiridinium core, resulting in UV-absorbing properties that are very unusual for a phospholipid. Consequently, favilipid A could inspire the development of a new class of molecules to be used as sunscreen ingredients. In addition, favilipid A inhibited by 58–48% three kinases (JAK3, IKKβ, and SYK) involved in the regulation of the immune system, suggesting a potential use for treatment of autoimmune diseases, hematologic cancers, and other inflammatory states.

## 1. Introduction

Marine natural products are considered a potentially important source of novel drug leads because they are structurally distinct in many ways from terrestrial natural products as a result of the different environment in which they evolved [1]. Modern methods of exploration and dereplication of chemical diversity from marine organisms, including LC-MS-based molecular networking [2,3], have made it possible to study previously intractable samples, such as large collections of microorganisms or small tissue samples from robotic deep-sea collections.

Marine sponges remain the second largest source of new marine natural products reported in 2020 [4], and the largest source of marine natural products reported so far [5]. The richness and variety of secondary metabolites present in marine sponges is related to their ability to host diverse microorganisms that are specifically and permanently associated with the sponge. Most of these microorganisms are currently unculturable [6], so the analysis of the sponge holobiome (with chemical or genomic means) is the only way to access this metabolomic diversity.

**Figure 1:**
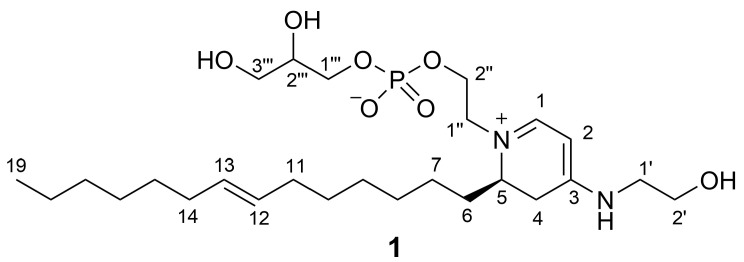
Structure of favilipid A (**1**).

After 50 years, the exploration of the chemistry of marine sponges is far from complete, and there are many species, including common ones, with undescribed chemical composition. Among them is *Clathria faviformis* [7], a sponge of the order Poecilosclerida usually found on vertical escarpments associated with Caribbean coral reefs. A combination of traditional dereplication strategies and feature-based molecular networking led to the discovery from *C. faviformis* of a new family of phospholipids, the favilipids. One of them, favilipid A (**1**) (Figure 1), was isolated and shown to possess an unprecedented structure characterized by a 4-aminodihydropiridinium core, exhibiting UV-absorbing properties that are very unusual in a phospholipid and that could inspire the development of a new class of sunscreen molecules.

## 2. Results

### 2.1. Extraction, LC-HRMS/MS Analysis, and Construction of the Molecular Network

A sample of *C. faviformis*, collected along the coast of Riding Rock Point in San Salvador Island (Bahamas Islands), was extracted with MeOH. The extract was dried and partitioned between H_2_O and EtOAc, and the resulting H_2_O layer was further partitioned with BuOH.

The combined EtOAc and the BuOH extracts, which represented the total organic extract of the sponge, were analyzed by liquid chromatography coupled with high-resolution tandem mass spectrometry (LC-HRMS/MS) on an LTQ Orbitrap instrument equipped with an ESI source, using an RP-18 column. The MS method involved four MS/MS scans after each full MS scan for the four most intense ions detected in the spectrum.

**Figure 2:**
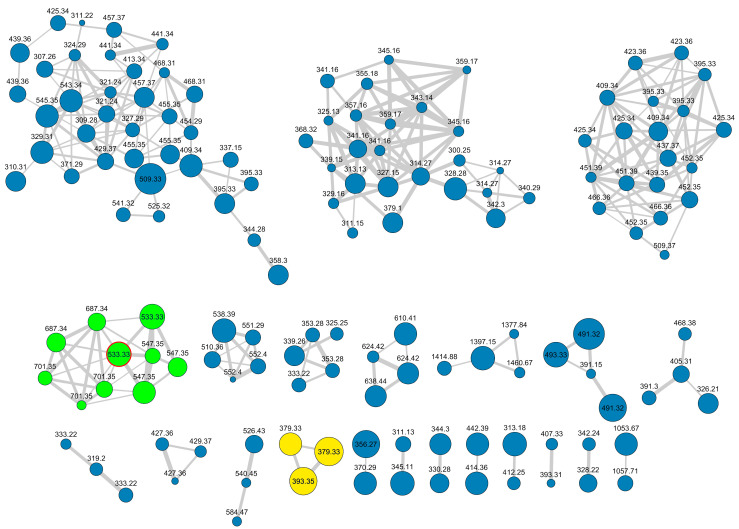
The feature-based molecular network of extracts from *C. faviformis*. The two clusters related to favilipids are colored in green and in yellow, respectively. The size of the nodes is related to the amounts of the metabolite. The node of favilipid A (**1**) is marked with red borders.

Following a workflow previously used for the isolation of stylissamide [8], raw LC-MS data were preprocessed using MZmine2 [9], resulting in an .mgf file containing MS/MS spectra and a .csv file containing quantitative data. Then, molecular networking parameters were fine-tuned using MetGem 1.3.6 [10], and the following set of parameters was found to generate the most informative network: minimum matched peaks 4, minimum cosine score value 0.60, maximum neighbor number 10, and maximum cluster size 1000. Next, processed LC-MS data were submitted to the GNPS website to produce the final version of the molecular network, using the Feature Based Molecular Networking workflow [11] and the aforementioned networking parameters. Finally, the resulting Feature-Based Molecular Network (Figure 2) was visualized using Cytoscape software [12], importing the relevant features from the quantitation file exported from MZmine2. In the network shown in Figure 2, the size of nodes is related with the intensity of the relevant ions, and therefore with the amounts of the relevant metabolites, whereas the thickness of edges connecting two nodes is related with the cosine score value between them.

Attention was focused on the cluster with the green nodes shown in Figure 2. The molecular formula of the compounds in the cluster, established from accurate mass of [M + H]^+^ ions and from the intensity of their isotope peaks, suggested the presence of at least one phosphorus in the structure as a common feature of all the metabolites in the cluster. The most abundant compound in the cluster (*m*/*z* 533.33), marked with red borders in Figure 2, was selected for isolation and chemical characterization.

### 2.2. Isolation and Structure Elucidation of Favilipid A (**1**)

The isolation of favilipid A (**1**) was achieved in three chromatographic steps. First, the EtOAc extract was subjected to normal-phase chromatography on a SiO_2_ column; the fraction eluted with 100% MeOH was further purified in two steps of reversed phase HPLC chromatography, yielding favilipid A as a pure compound (**1**). The weight of the isolated favilipid A (**1**) was determined using an NMR quantitation method based on integration of solvent ^13^C satellite peaks [13], and determined to be as little as 12.8 μg, which was enough for complete chemical characterization.

**Figure 3:**
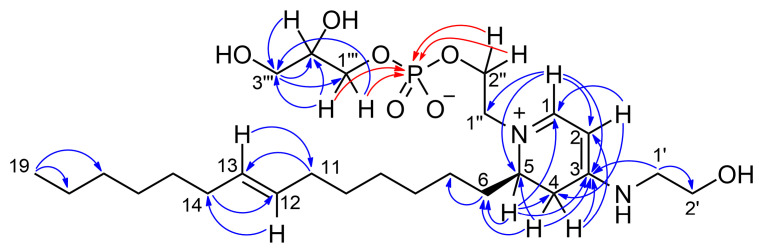
Most significant ^1^H-^13^C (blue arrows) and ^1^H-^31^P (red arrows) HMBC correlations of favilipid A (**1**).

The high-resolution ESI mass spectrum of favilipid A (**1**) showed an [M + H]^+^ peak at *m*/*z* 533.3335 (Figure S1), in accordance with the molecular formula C_26_H_49_N_2_O_7_P (calcd. value for C_26_H_50_N_2_O_7_P is *m*/*z* 533.3350), accounting for four unsaturations. The presence of a P atom in the molecule was confirmed by a ^31^P-^1^H HMBC spectrum, showing coupling of a ^31^P nucleus resonating at δ 0.7 with four methylene protons at δ 4.10, 4.00, 3.89, and 3.84 (Figure 3 and Figure S8). These data suggested the presence of a phosphodiester function in the molecule. The ^1^H NMR spectrum of compound **1** (Table 1 and Figure S2) showed signals of a monounsaturated alkyl chain (δ 5.39, 2H, vinylic protons; 1.98, 4H, allylic protons; 1.38–1.28, broad band, methylene protons; and 0.90, 3H, terminal methyl group) suggesting it to be a phospholipid. In addition, the ^1^H NMR spectrum showed a number of signals between δ 4.10 and 3.46, suggesting the presence of glycerol and/or ethanolamine units. Specifically, combined analysis of the COSY (Figure S3), TOCSY (Figure S4), and HSQC (Figure S6) 2D NMR spectra identified one glycerol residue (1‴: δ_C_ 67.7, δ_H_ 3.89 and 3.84; 2‴: δ_C_ 72.6, δ_H_ 3.75; 3‴: δ_C_ 63.9, δ_H_ 3.59, and 3.54) and two ethanolamine residues (1′: δ_C_ 47.4, δ_H_ 3.46 (2H); 2′ δ_C_ 60.3, δ_H_ 3.75 (2H); 1″: δ_C_ 56.6, δ_H_ 3.72 and 3.66; 2″: δ_C_ 64.6, δ_H_ 4.10 and 4.00). The latter ethanolamine residue was connected with the glycerol residue through the phosphodiester bond discussed above.

**Figure 4:**
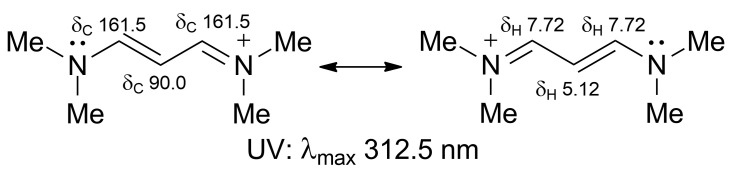
^1^H NMR [15],^13^C NMR [16], and UV [17] spectral data of a simple trimethine cyanine dye show similarity with those of favilipid A (**1**).

While major phospholipid classes show no UV absorption above 220 nm, the UV spectrum of compound **1** was quite peculiar, in that it showed a strong absorption band at 344 nm (ε = 15,000) and little or no absorption at shorter wavelengths until 210 nm. This suggested the presence of a conjugated pi system in the molecule. In addition to the *sp*^2^ carbons of the monounsaturated alkyl chain, ^13^C NMR data revealed the presence of two CH (δ 159.3, C-1 and 86.4, C-2) and one C (δ 165.4, C-3) *sp*^2^ carbon atoms. Their peculiar chemical shifts and their HMBC correlations with the relevant protons H-1 (δ 7.63) and H-2 (δ 5.28) (Figure 3 and Figure S7) suggested a polyunsaturated system similar to that of a trimethine cyanine dye [14] (Figure 4), i.e., a three-methine chain linking two terminal tertiary nitrogen atoms.

The HMBC correlation (Figure 3) of H-1 with C-1″ (a carbon atom belonging to one of the two ethanolamine residues) and that of H_2_-1′ (a proton belonging to the other ethanolamine residue) with C-3 strongly supported this hypothesis. The non-protonated carbon atom C-3 was also linked to a methylene group (C-4) showing strongly diastereotopic protons (δ 3.29, dd, *J* = 17.0 and 7.1 Hz, H-4a; δ 2.67, br. d, *J* = 17.0 Hz, H-4b). This was deduced by the HMBC correlations of H-2 with C-4, of H-4a with C-2 and C-3, and of H-4b with C-3. In turn, C-4 was linked to a methine group (δ 56.1, C-5 and δ 3.87, H-5), as shown by the COSY correlation of H-5 with H-4a and the HMBC correlations of H-5 with C-4 and C-3). Finally, the HMBC correlations of H-1 with C-5 and of H-5 with C-1 showed that C-5 was linked to the N atom also linked to C-1, to close a 4-aminodihydropyridinium ring. The planar structure of favilipid A (**1**) was completed by connecting the methine carbon C-5 and the monounsaturated alkyl chain via the methylene group CH_2_-6 (δ_C_ 28.9, C-6 and δ_H_ 1.67, H_2_-6) based on the HMBC correlations of H-5 and H-4a with C-6.

The length of the monounsaturated alkyl chain linked to C-5 was determined to be C_14_ from the molecular formula; the *E* configuration of the double bond in the chain was determined from the ^13^C chemical shift of the allylic carbons C-11 and C-14 (δ 33.5), which was appropriate for the allylic methylene groups of an *E* double bond in an unbranched chain (the expected value for the *Z* isomer is shielded by about 5 ppm) [18].

**Figure 5:**
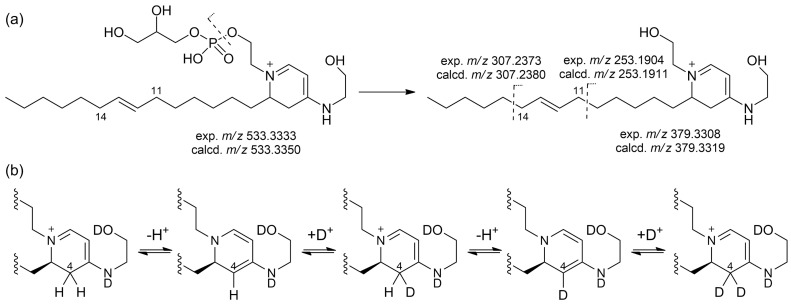
(**a**) An MS^3^ experiment allowed location of the double bond on the alkyl chain of favilipid A (**1**). (**b**) The slow deuterium exchange of favilipid A (**1**) in CD_3_OD.

The location at position 12 of the double bond on the monounsaturated alkyl chain was established on the basis of a high-resolution MS^3^ spectrum, analogous to the fragmentation observed in the MS^3^ spectrum of the sphingolipids reported in ref. [19]. The most prominent peak in the MS^2^ spectrum of favilipid A (**1**) was a fragment ion at *m*/*z* 379.3312, corresponding to the neutral loss of dehydrated glycerol phosphate, i.e., MW 154.0031. Further fragmentation of this ion in an MS^3^ experiment yielded two fragment ions at *m/z* 307.2373 and 253.1904, corresponding, respectively, to the two possible allylic cleavages of the alkyl chains (Figure 5a).

The planar structure of favilipid A (**1**) was confirmed by fragment ions in the MS^2^ and MS^3^ spectra, which could be easily rationalized by the proposed structure (Figure S11), and by the correlations displayed by the ROESY spectrum (Table 1 and Figure S5). Further confirmation of structure **1** came from the slow exchange with deuterium of both protons at C-4 observed when the compound was dissolved in CD_3_OD (the time constant of the process was not measured precisely, but its order of magnitude was several days at room temperature). The most likely mechanism for the exchange is reversible deprotonation of the *N*-alkyl-4-amino-2,3-dihydropyridinium system of favilipid A to generate a *N*-alkyl-4-amino-1,2-dihydropyridine (Figure 5b).

**Figure 6:**
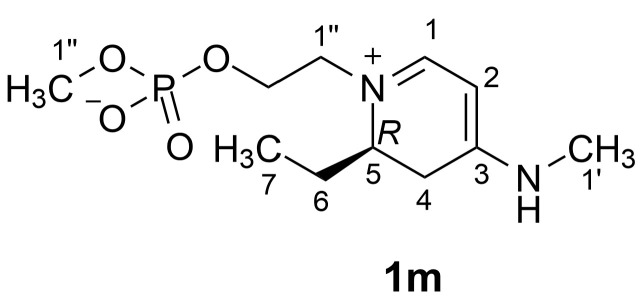
Structure of the simplified model compound **1m** of favilipid A used for prediction of the ECD spectrum.

Two stereogenic centers are present in favilipid A (**1**), C-5 in the dihydropyridine ring and C-2‴ in the glycerol residue. While the configuration at C-2‴ could not be established by chemical derivatization because the small sample available had to be preserved for biological assays, the configuration at C-5 was determined non-destructively by DFT prediction of the electronic circular dichroism (ECD) spectrum. DFT calculations were performed using the truncated model compound **1m**, because the full molecule is very flexible, especially in the alkyl chain and in the phosphoglycerol unit, and therefore exists in an untreatably high number of conformations. Moreover, the carbinol groups of the glycerol residue and of the eastern ethanolamine residue, and most of the carbon atoms of the alkyl chain are far enough from the chromophore that they do not significantly affect the ECD spectrum it produces [20]. On the other hand, maintaining the phosphodiester function in the model compound was judged to be essential for an accurate prediction, as it ensures the electroneutrality of the molecule and is expected to experience strong electrostatic interactions with the positively charged nitrogen atom in the dihydropyridine ring. The *R* enantiomer of model compound **1m** was randomly chosen for calculations (Figure 6).

Systematic conformational search identified 16 low energy conformers within 3 kcal/mol from the lowest energy conformer; their UV and ECD spectra were predicted using Time Dependent DFT (TDDFT) at the B3LYP/6-311+G(2d,p)/SMD(MeOH) level of theory, and average UV and ECD spectra were obtained from them with the program SpecDis [21] using Boltzmann statistics. The predicted UV spectrum showed only one π→π* transition above 250 nm, in accordance with the UV spectrum of favilipid A (**1**) (Figure S9). The predicted absorption maximum (307 nm) was considerably blueshifted compared to the experimental value (344 nm), but this is a well-known problem with TDDFT prediction of UV spectra of cyanine dyes [22]. The predicted ECD spectrum was of course also blueshifted, with a predicted negative absorption band at 307 nm, compared to the experimental negative absorption band at 351 nm (Δε = –1.3). The matching sign of the predicted and experimental ECD spectra (Figure S10) showed that the *R* enantiomer of model compound **1m** chosen for calculation matched the 5*R* absolute configuration of natural favilipid A (**1**).

### 2.3. Other Favilipids

While favilipid A (**1**) was the only compound obtained in pure form at this time, examination of the relevant cluster in the molecular network (Figure 2) made clear that a large set of analogous favilipids are present in the extract of *C. faviformis*. Consideration of their MS, MS^2^, and MS^3^ spectra provided some preliminary information of their structures.

**Figure 7:**
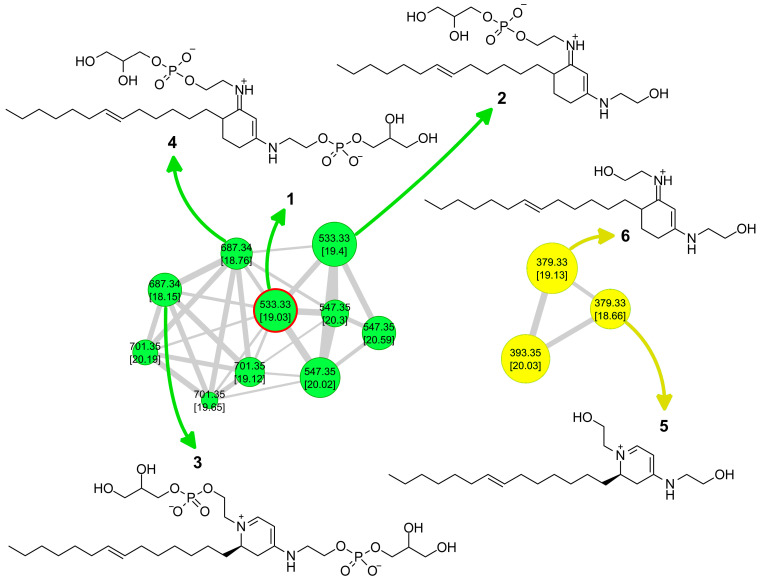
Putative structures of additional favilipids identified by molecular networking. Each node is labeled with the relevant *m/z* and, in square brackets, the retention time in min. Nodes with *m/z* 701.35, 547.35, and 393.35 are higher homologues of, respectively, nodes with *m/z* 687.33, 533.33, and 379.33.

In addition to the node of favilipid A (**1**), the cluster contains another node with the same *m*/*z* 533.33 and different retention time, characterized by similar fragmentation pattern in the 533.33→MS^2^ spectrum (base peak at *m/z* 379.33, loss of phosphoglycerol residue), but a different fragmentation of the latter ion in the 533.33→379.33→MS^3^ spectrum (Figure S12). A plausible, but purely speculative structure for this, compound is proposed as structure **2** (Figure 7). Again, structure **2** contains a cyanine-like chromophore, which in this case resembles that of the widespread UV-protecting marine compounds, mycosporine-like amino acids (see Section 3 below).

The two nodes with *m*/*z* 687.33 are consistent with the formula C_29_H_57_O_12_N_2_P_2_, i.e., one additional phosphoglycerol residue (C_3_H_7_O_5_P) compared to **1**. These are analogues of **1** and **2**, in which both the OH groups at C-2′ and C-2″ are linked to glycerol molecules through phosphodiester bonds (structures **3** and **4**, respectively). Structures **3** and **4** are supported by their respective 687.33→MS^2^ spectra (base peak at *m/z* 533.33, loss of one phosphoglycerol residue) and 678.33→533.33→MS^3^ spectra (base peak at *m/z* 379.33, loss of two phosphoglycerol residues).

Another cluster in the network, also shown in Figure 7, is related to the favilipids. It contains two nodes with *m/z* 379.33, which can be assigned to two analogues of **1** and **2**, in which no phosphoglycerol residue is present (structures **5** and **6**, respectively). This is confirmed by the fact that the 379.33→MS^2^ spectrum of compound **5** is identical to the 533.33→379.33→MS^3^ spectrum of compound **1**, and the 379.33→MS^2^ spectrum of compound **6** is identical to the 533.33→379.33→MS^3^ spectrum of compound **2**.

Finally, the nodes with *m*/*z* 547.35, 701.35, and 393.35 in the two clusters can be assigned to higher homologues of, respectively, either **1** or **2**, either **3** or **4**, and either **5** or **6**. Based on their respective MS^2^ and MS^3^ spectra, the additional CH_2_ appears to be located on the alkyl chains.

Pending the final determination of putative structures **2–6**, no trivial names were assigned to them.

### 2.4. Kinase Activity Assays

**Figure 8:**
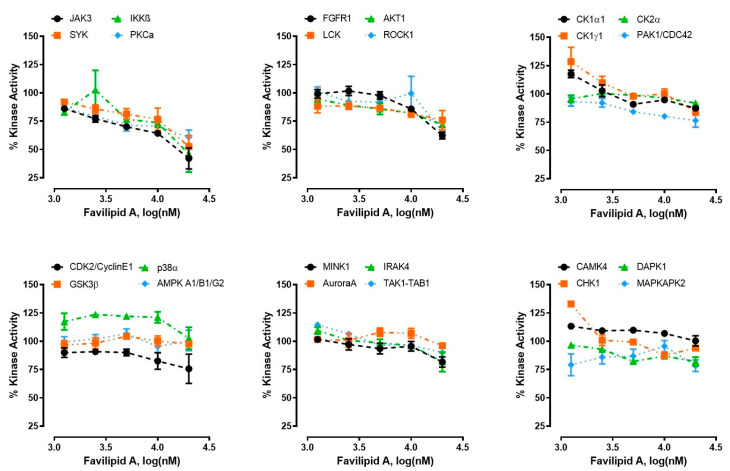
Favilipid A (**1**) was tested against 24 kinases in 5-point inhibitor dose response. The dose–response curves show the percentages of kinases activity and represent the averages of two replicates.

The activity of favilipid A (**1**) was screened at 5-point concentration (1.25, 2.5, 5, 10, and 20 µM) against a general panel of 24 kinases representative of the human kinome in a functional kinase assay, performed by measuring the conversion of ATP to ADP. The results (Figure 8) demonstrated that favilipid A is an inhibitor of the kinases JAK3 (maximum inhibitory effect 58%), IKKβ (54%), and SYK (48%) with IC_50_ in the low-μM range (Table 2) and, to a lesser extent, of the kinases PKCα (40%) and FGFR1 (38%). In contrast, other kinases were either not inhibited or weakly inhibited (specifically, AKT1, LCK, PAK1, and ROCK1 were inhibited about by 25–30%)

## 3. Discussion

Analysis by high-resolution LC-MS^2^ and molecular networking of the extracts of the marine sponge *Clathria faviformis* resulted in the discovery of a new family of UV-absorbing phospholipids, the favilipids. One of them, favilipid A (**1**), was isolated in pure form and structurally characterized.

This study confirmed the value of molecular networking in the discovery of new natural products. However, the small amounts (12.8 μg) of favilipid A (**1**) that could be isolated also illustrated an important issue with molecular network-based identification of new natural products and, more generally, with MS based identification of new natural products. Even though the node with *m*/*z* 533.33 was one of the largest in the network, the amount of corresponding metabolite favilipid A (**1**) was not as large as expected. It is certainly true that the intensity of an LC-MS peak is proportional to the amounts of the compounds in the extract, but this intensity also depends on the ionization efficiency in the ESI source, which can be dramatically different from compound to compound. Favilipid A (**1**), a zwitterionic compound, was apparently ionized very efficiently, and gave rise to large LC-MS peak even though it was present in a low concentration in the extract. This issue does not affect the usefulness of molecular networking for the fast identification of new natural products in a complex extract, but must be taken into account when analyzing a network.

**Figure 9:**
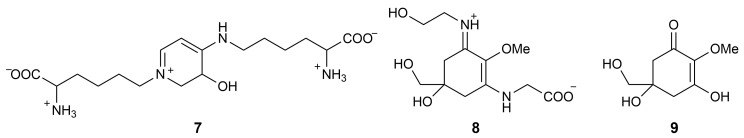
Natural products structurally related to favilipids: K2P (**7**) from human lens protein digests, asterina-330 (**8**), an example of mycosporine-like amino acids (MAAs), and 4-deoxygadusol (**9**), the common biogenetic precursor of MAAs.

Favilipid A (**1**) has no close analogues among natural products. Like many phospholipids, it contains two ethanolamine and one glycerol units, but the core of the molecule is built on a C_19_ unbranched alkyl chain with an ω-7 trans double bond with no obvious biosynthetic origin. The most similar natural compound reported in the literature is K2P (**7**) [23], a compound isolated from the enzymatic digest of human lens protein (Figure 9). It shares the same 4-aminodihydropyridinium chromophore as in favilipid A, but is otherwise a very different molecule, appearing as a cross-linking product between two lysine residues and a pentose.

An important class of molecules, which are related to favilipid A because of the presence of a cyanine-like chromophore and because of their UV-protecting properties, are the mycosporine-like amino acids (MAAs) [24]. MAAs (e.g., asterina-330, **8**) are water-soluble compounds characterized by a 3-aminocyclohexenimine core, originating from condensation of the common precursor 4-deoxygadusol (**9**) with different amino acids or amino alcohols [25]. MAAs are an important class of UV-protecting natural molecules, and are attracting a great deal of interest as sunscreens and cosmetic ingredients [24].

There is an urgent need for safer, biodegradable, and environmentally friendly sunscreen ingredients. Because of its relatively simple structure, favilipid A could inspire the development of an alternative, more lipophilic class of compounds for these purposes.

Even though the production of favilipid A by *C. faviformis* or one of its symbionts may be related to its potential UV-protecting properties, a different role for this compound cannot be excluded. In light of this, the activity of favilipid A as a potential inhibitor of kinases was evaluated. Kinases have emerged as promising clinical targets; they play key roles in different biological processes, such as signal transduction, cell proliferation and differentiation, metabolic processes, apoptosis, etc. Activation of mutations in kinases have been linked to a number of disorders and diseases, most notably cancers [26]. Remarkably, favilipid A (**1**) inhibited (58–48%) three kinases (JAK3, IKKβ, and SYK) involved in regulation of the immune system. Inhibitors of these kinases are under consideration for treatment of immune disorders [27,28,29], and one inhibitor of JAK3, tofacitinib, is in clinical use for treatment of rheumatoid arthritis [30]. Therefore, favilipid A could inspire the design of a new class of kinase inhibitors to be used for treatment of autoimmune diseases, hematologic cancers, and other inflammatory states.

The present study paves the way for a substantial amount of future research. We intend to isolate the other favilipid identified from the molecular network and determine/confirm their structure; to investigate the biosynthetic origin of favilipids, taking advantage of the availability of a definite structure for the isomer **2** of favilipid A; to verify the possible presence of favilipids in organisms other than *C. faviformis*; and to deepen our knowledge of the biological activity of favilipid A and its analogues.

## 4. Materials and Methods

### 4.1. General Experimental Procedures

UV spectra were recorded on a Jasco V-530 spectrophotometer (Jasco Europe s.r.l., Cremella, Italy); ECD spectra were recorded on a Jasco 715 spectropolarimeter using 1 cm cuvettes; ^1^H NMR and 2D NMR experiments were carried out at 700 MHz on a Bruker Avance Neo spectrometer (Bruker BioSpin Corporation, Billerica, MA, USA) equipped with a cryoprobe for standard 5 mm tubes; ^1^H and ^13^C chemical shifts were referenced to the residual solvent signal (CD_3_OD, δ_H_ 3.31, δ_C_ 49.0); ^31^P chemical shifts were referenced to aqueous 85% *w*/*w* H_3_PO_4_ (δ_P_ = 0 ppm) as an external standard. The multiplicity-edited HSQC spectra was optimized for ^1^*J*_CH_ = 145 Hz and the HMBC experiments for ^2,3^*J*_CH_ = 8.0 Hz and ^2,3^*J*_PH_ = 8.0 Hz. Through-space ^1^H connectivities were evidenced using a ROESY experiment with a mixing time of 200 ms. High-resolution ESI-MS and HR-ESI-HPLC experiments were recorded on a Thermo LTQ Orbitrap XL mass spectrometer (Thermo Fisher Scientific Inc., Waltham, MA, USA) combined to a Thermo U3000 HPLC system. High-performance liquid chromatography (HPLC) separations were achieved on an Agilent 1260 Infinity Quaternary LC apparatus (Agilent Technology, Cernusco sul Naviglio, Italy), equipped with a diode-array detector (DAD).

### 4.2. Collection, Extraction, and Isolation

The specimen of the sponge *Clathria faviformis*, analyzed in this study, was collected at 30 m depth by scuba diving along the coast of Riding Rock Point, in San Salvador, Bahamas, during a ship-based expedition in 2003. Once collected, the sponge was immediately frozen and kept at −20 °C until extraction. The frozen sponge (173.7 g wet weight) was chopped into small pieces and kept at −20 °C for 1h prior to lyophilization. After a 34 h lyophilization, the sponge (39.6 g dry weight) was extracted at room temperature with MeOH (3 × 0.7 L). The yellow colored MeOH extract was concentrated under a vacuum, until a volume of 500 mL of total extract remained. Then, 250 mL of H_2_O was added and the H_2_O-MeOH extract was concentrated under a vacuum reaching the volume of 500 mL. A second aliquot of 250 mL of H_2_O was added, and the extract was concentrated under a vacuum until 650 mL remained. The H_2_O-MeOH extract was first partitioned with H_2_O-saturated EtOAc. The resulting H_2_O layer was then partitioned with *n*-BuOH. The EtOAc (1.3 g) and *n*-BuOH (1.8 g) extracts were used for LC-MS/MS analysis. The EtOAc extract was chromatographed by normal-phase chromatography on a SiO_2_ column, using the elution step gradient *n*-hexane/EtOAc (1:1), *n*-hexane/EtOAc (2:8), 100% EtOAc, EtOAc/MeOH (9:1), EtOAc/MeOH (1:1), and 100% MeOH. The fraction eluted with 100% MeOH (106.8 mg) was subjected to two steps of reversed-phase HPLC separation. Initial HPLC purification was achieved on a Luna C18 column (250 × 10 mm, 10 μm) (Phenomenex, Torrance, CA, USA) [Eluent A: 0.1% HCOOH in H_2_O; eluent B: ACN; gradient program: 50% B 1 min, 50% → 80% B over 15 min, 80% → 95% B over 1 min, 95% B 5 min; flow rate 5 mL min^–1^, wavelength 330 nm]. Further purification of the fraction containing compound **1** was performed on a Phenomenex Luna C18(2) column (250 × 4.6 mm, 5 μm) [Eluent A: 0.1% HCOOH in H_2_O; eluent B: MeOH; gradient program: 30% B 1 min, 30% → 100% B over 20 min,100% B 10 min; flow rate 1 mL min^–1^, wavelength 330 nm] to afford a fraction (*tR* = 22 min, 12.8 μg) composed of pure favilipid A (**1**).

*Favilipid A* (**1**): white amorphous solid, HRESIMS (positive ion mode, MeOH) *m/z* 533.3335 [M+H]^+^_,_ (C_26_H_50_O_7_N_2_P calcd. 533.3350). ^1^H and ^13^C NMR (CD_3_OD): Table 1; UV (MeOH): λ_max_ (ε) 344 nm (15,000); ECD (MeOH): λ_max_ (Δε) 351 nm (−1.3).

### 4.3. LC-HRMS and LC-HRMS/MS

All LC-HRMS and LC-MS/MS analyses were performed on a Thermo LTQ Orbitrap XL high-resolution ESI mass spectrometer coupled to a Thermo U3000 HPLC system. A Phenomenex Kinetex C18(2) column (100 mm × 2.10 mm, 5 μm) kept at 25 °C and an elution gradient with 0.1% HCOOH in H_2_O (eluent A) and MeOH (eluent B) and a flow rate of 200 μL/min were used. The gradient was as follows 30% B for 1 min, 30–100% B over 20 min, and 100% B for 10 min. The volume injected was set at 5 µL. Mass spectra were acquired in positive ion detection mode, with resolution set to 60.000 in the range of *m/z* 300–2000. The parameters used to perform the analysis were a spray voltage of 4.80 kV, a capillary temperature of 285 °C, a sheath gas rate of 32 units N_2_ (ca. 320 mL/min), and an auxiliary gas rate of 15 units N_2_ (ca. 150 mL/min). Data were recorded with data-dependent acquisition (DDA) mode, in which the four most intense ions in the full-scan mass spectrum were subjected to high resolution tandem mass spectrometry (HRMS/MS) analysis. HRMS/MS scans were achieved for selected ions with collision induced dissociation (CID) fragmentation, an isolation width of 6.00 Da, a normalized collision energy of 35 units, an activation Q of 0.250 units, and an activation time of 30 ms. Mass data were analyzed using the Thermo Xcalibur software version 2.2.

### 4.4. LC-HRMS/MS Data Processing and Molecular Networking

First, LC-HRMS/MS spectra were converted from raw file to mzXML file format using MSconvert, from the ProteoWizard suite [31], and then processed in batch mode with the software MZmine 2.53 [9], as follows. Mass detection of raw data files was performed on centroid data by keeping the noise level at 8.0E+3. FTMS shoulder peaks were removed applying the Lorentzian extended peak model at a resolution of 30,000. The detection of chromatograms was performed using the ADAP chromatogram builder setting at least 5 consecutive scans in the chromatogram, a minimum height of 50,000, a group intensity threshold of 5000, and an *m/z* tolerance of 0.01 Da or 10 ppm, whatever was smaller, among data points in sequential scans to be merged in the same chromatogram. Spectral deconvolution was performed using the local minimum search algorithm, setting a chromatographic threshold for removing noise of 10%, the search of minimum in a range of 0.15 min, the minimum relative height and the minimum absolute height were, respectively, set at 5% and 100,000, the minimum ratio between peak top intensity and side data points at 1.3, and maximum peak duration at 10 min. Peak alignment was performed with the Join aligner algorithm using the following parameters: *m/z* tolerance at 0.005 (or 5 ppm), absolute RT tolerance 0.3 min, and a score weight for *m/z* and for retention time set at 80 and 20, respectively. Common adducts were recognized and filtered out setting the maximum difference in the retention time at 0.1 min, the maximum *m/z* difference between peaks at 0.001 Da or 5 ppm, and the maximum relative adduct peak height at 100%. Peaks without an associated MS/MS spectrum were filtered out from the peak list. The feature list spectra were then exported into a .mgf file, and the table of quantification was exported as a .csv file.

Molecular networking parameters were first optimized with MetGem software 1.3.6 [10]. The *m/z* tolerance was set at 0.02 Da; in the molecular network, the edges with a cosine score higher than 0.6 and with more than 4 matched peaks were combined into consensus spectra; the maximum number of neighbor nodes for one single node was set at 10, and the maximum size of nodes allowed in a single connected network was set to 1000. The processed LC-MS data were submitted to the GNPS website using the Feature Based Molecular Networking workflow [11] and the aforementioned networking parameters to produce the final version of the molecular network, which was visually displayed with Cytoscape 3.7.1 [12].

### 4.5. Computational Details

A model of compound **1m** (the *R* enantiomer was randomly chosen) was built using the Insight II/Discover package (BIOVIA, San Diego, CA, USA). Conformational search with the grid search algorithm was performed using the same software (Search_Compare module). The dihydropyridine ring may exists in two opposite half-chair conformations, and they were both considered in two independent runs of systematic search. The C-5/C-6, C-1″/N, C-1″-C2″, and C-2″/O torsions were systematically changed by 120°, whereas the C-3′/N torsion was set either at 0° or 180° to preserve the planarity of the conjugated system. The search resulted in 108 conformers, 16 of which presented clear steric clashes and were not considered further.

The remaining 92 conformers were optimized quantum-mechanically using DFT at the B3LYP/6-31G+(d,p)/SMD(MeOH) level with the Gaussian 16 program (Revision C.01, Gaussian Inc., Wallingford CT, USA). The UV and ECD spectra of the 16 low energy conformers (energy within 3 kcal/mol from the lowest energy conformer) were then predicted using Time Dependent DFT (TDDFT) at the B3LYP/6-311+G(2d,p)/SMD(MeOH) level of theory. The Cartesian coordinates of these conformers are reported in Table S1.

The UV and ECD spectra of each conformer were generated using the program SpecDis [21] and a half-band width σ = 0.35 eV, and average UV and ECD spectra were obtained from them using Boltzmann statistics (Figure S10). All the predicted UV and ECD spectra in Figure S10 are shifted by +37 nm to match the predicted (307 nm) and experimental (344 nm) UV maxima.

### 4.6. Kinase Activity Assay

Kinase activity assay was performed using the Kinase Selectivity Profiling System: General Panel + ADP-Glo™ Kinase Assay (Promega, Madison, WI, USA). Favilipid A was assayed at 5-point concentration (1.25, 2.5, 5, 10, and 20 µM) against 24 kinases representative of the human kinome. The assay was performed according to the manufacturer’s instructions. Results were expressed as percentage of kinase activity and represent the averages of two replicates ± SEM (Standard Error Mean). The IC_50_ values were calculated using nonlinear regression with normalized dose–response fitting using Prism software (GraphPad 8 Software, San Diego, CA, USA).

## Figures and Tables

**Table 1:** NMR data of favilipid A (**1**) (700 MHz, CD_3_OD).

Pos.	δ_C_, Type		δ_H_, Mult (*J* in Hz)	ROESY	^13^C and ^31^P HMBC
1	159.3, CH		7.63, d (6.6)	H-2, H1″a	C-1, C-3, C-5, C-1″
2	86.4, CH		5.28, d (6.6)	H-1, H_2_-1′	C-1, C-4
3	165.4, C		-		
4	32.4, CH_2_	a	3.29, dd (17.0, 7.1)	H-4b	C-3, C-5, C-6
		b	2.67, br. d (17.0)	H-4a, H-5	C-2, C-3
5	56.1, CH		3.87, br. quartet (7.1)	H-4b, H_2_-6	C-1, C-3, C-4, C-6
6	28.9, CH_2_		1.67, quartet (7.1)	H-5, H-1″b	C-4, C-7, C-8
7	26.4, CH_2_	a	1.38, m		
		b	1.32, m		
8, 9, 16	30.1, CH_2_		1.34–1.29, m		
10	30.5, CH_2_		1.34, m	H-12	
11	33.5, CH_2_		1.98, m	H-13	C-10, C-12, C-13
12	131.4, CH		5.39, m	H_2_-10, H-14	C-11, C-14
13	131.4, CH		5.39, m	H_2_-13, H-11	C-11, C-14
14	33.5, CH_2_		1.98, m	H-12	C-12, C-13, C-15
15	30.5, CH_2_		1.34, m	H-13	
17	32.9, CH_2_		1.28, m		
18	23.6, CH_2_		1.31, m	H_3_-19	
19	14.3, CH_3_		0.90, t (7.0)	H_2_-18	C-17, C-18
1′	47.4, CH_2_		3.46, t (5.3)	H-2, H_2_-2′	C-3, C-2′
2′	60.3, CH_2_		3.75, t (5.3)	H_2_-1′	
1″	56.6, CH_2_	a	3.72, ddd (14.6, 7.8, 3.3)	H-1, H-1″b, H-2″b	
		b	3.66, ddd (14.6, 5.0, 3.6)	H_2_-6, H-1″a, H-2″b	
2″	64.6, CH_2_	a	4.10, m	H-2″b	P
		b	4.00, m	H-1″a, H-1″b, H-2″a	P
1‴	67.7, CH_2_	a	3.89, m	H-1‴b	C-3‴, P
		b	3.84, m	H-1‴a	C-2‴, C-3‴, P
2‴	72.6, CH		3.75, m	H-3‴b	C-1‴, C-3‴
3‴	63.9, CH_2_	a	3.59, dd (11.4, 5.3)	H-3‴b	C-1‴, C-2‴
		b	3.54, dd (11.4, 5.7)	H-2‴, H-3‴a	C-1‴, C-2‴

**Table 2:** Maximum inhibitory effect (at the highest concentration used, expressed as a percentage) of favilipid A (**1**) on kinase activities and corresponding IC_50_ values (concentrations eliciting 50% of the maximum response) with 95% confidence intervals (CI).

Kinase	% Inhibition	IC_50_ (μM)	95% CI (μM)
JAK3	58%	3.5	2.0–5.9
IKKβ	54%	6.0	1.4–77.8
SYK	48%	8.3	4.9–13.8
PKCα	40%	3.3	2.1–5.0
FGFR1	38%	10.7	8.8–12.9

## Data Availability

The molecular network discussed in this paper is available on GNPS at https://gnps.ucsd.edu/ProteoSAFe/status.jsp?task=188141d302e74051a0ad98a5a63aba9f.

## Supplementary Materials

The following supporting information can be downloaded at: https://www.mdpi.com/article/10.3390/md21020058/s1. Spectra of favilipid (**1**): Figure S1: ESI mass spectrum; Figure S2: ^1^H-NMR spectrum; Figure S3: COSY spectrum; Figure S4: TOCSY spectrum; Figure S5: ROESY spectrum; Figure S6: ^13^C-HSQC spectrum; Figure S7: ^13^C-HMBC spectrum; Figure S8: ^31^P-HMBC spectrum; Figure S9: UV and ECD spectra; Figure S10: UV and ECD spectra of individual conformers and predicted average spectra of favilipid A (**1**) in MeOH; Figure S11: Interpretation of MS^2^ and MS^3^ spectra of favilipid A (**1**); Figure S12: MS^2^ and MS^3^ spectra of favilipid A (**1**) and of its isomer **2**; Table S1: Cartesian coordinates of the 16 low-energy conformers of the model compound **1m** of favilipid A.
