# Supplementary material for: Molecular Networking Revealed Unique UV-Absorbing Phospholipids: Favilipids from the Marine Sponge Clathria faviformis
Source: Mar Drugs. 2023 Jan 18;21(2):58. doi: 10.3390/md21020058 (PMC9965855; doi:10.3390/md21020058)
Supplement: Supplementary file 1 [file marinedrugs-21-00058-s001.zip › marinedrugs-2159895-supplementary.pdf]

Supplementary Material

Figure S1. Positive ion mode high-resolution ESI mass spectrum of favilipid A (**1**).

Figure S2. ^{1}H-NMR spectrum of favilipid A (**1**) (700 MHz, CD_3OD).

Figure S3. COSY spectrum of favilipid A (**1**) (700 MHz, CD_3OD).

Figure S4. TOCSY spectrum of favilipid A (**1**) (700 MHz, CD_3OD)

Figure S5. ROESY spectrum of favilipid A (**1**) (700 MHz, CD_3OD).

Figure S6. ^{13}C-HSQC spectrum of favilipid A (**1**) (700 MHz, CD_3OD).

Figure S7. ^{13}C-HMBC spectrum of favilipid A (**1**) (700 MHz, CD_3OD).

Figure S8. ^{31}P-HMBC spectrum of favilipid A (**1**) (700 MHz, CD_3OD).

Figure S9. UV and ECD spectra of favilipid A (**1**) in MeOH.

Figure S10. UV and ECD spectra of individual conformers and predicted average spectra of favilipid A (**1**) in MeOH

Figure S11. Interpretation of MS^2 and MS^3 spectra of favilipid A (**1**).

Figure S12. MS^2 and MS^3 spectra of favilipid A (**1**) and of its isomer **2**.

Table S1. Cartesian coordinates of the 16 low-energy conformers of the model compound **1m** of favilipid A.

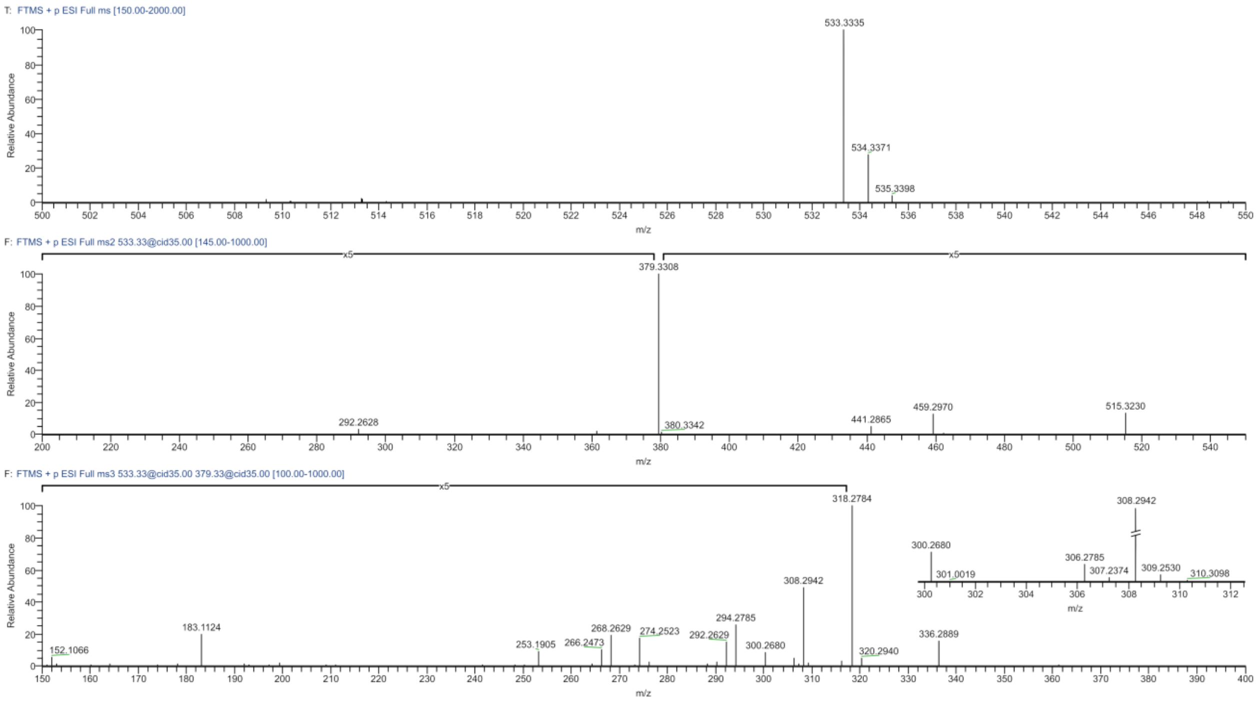

Figure S1. High resolution ESI MS data of favilipid A (**1**): MS spectrum (top panel), 533.33→MS^2 spectrum (middle panel), and 533.33→379.33→MS^3 spectrum (bottom panel).

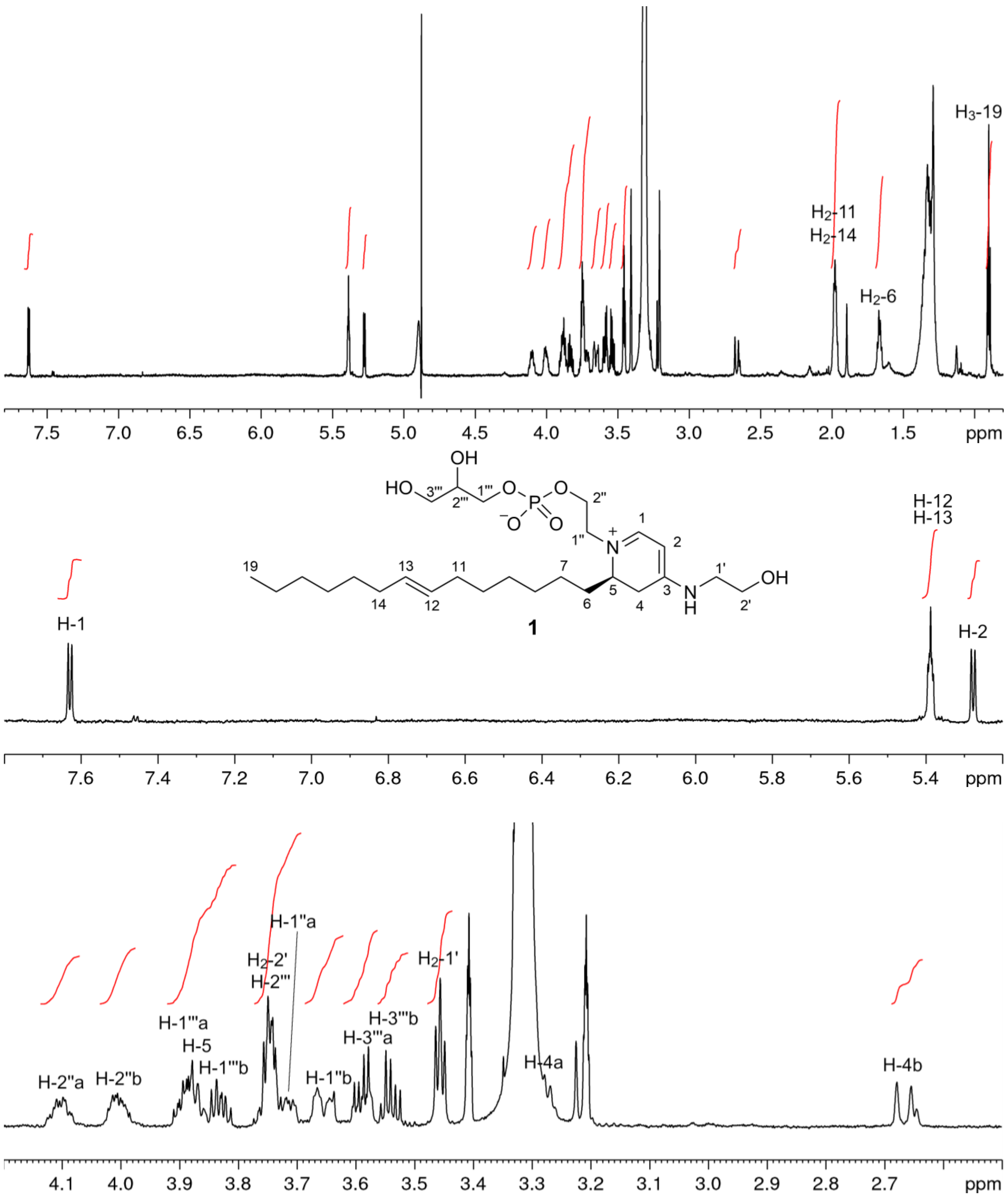

Figure S2. ^{1}H-NMR spectrum of favilipid A (**1**) (700 MHz, CD_3OD): full spectrum (top panel), low-field expansion (middle panel), middle-field expansion (bottom panel)

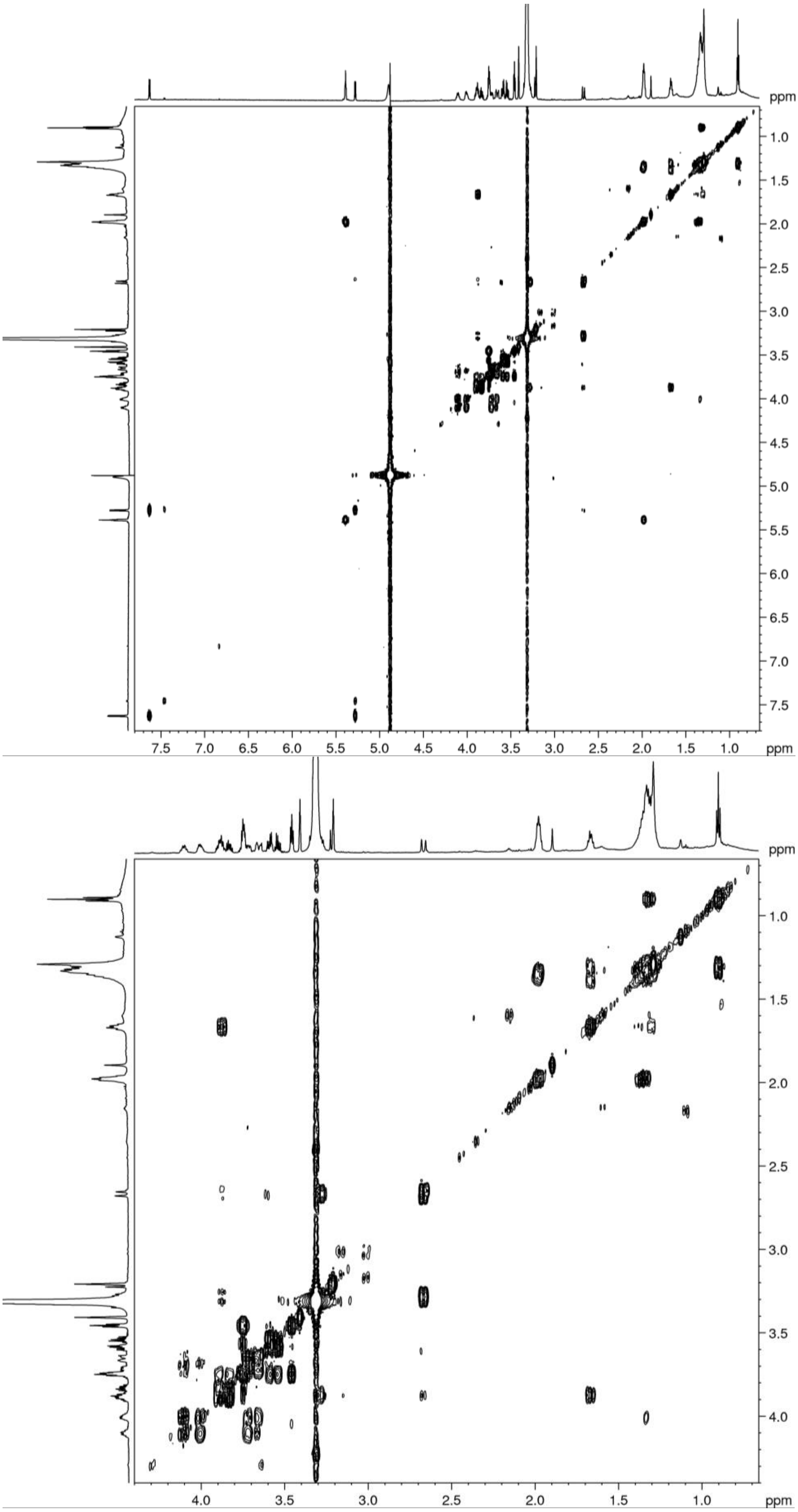

Figure S3. COSY spectrum of favilipid A (**1**) (700 MHz, CD_3OD): full spectrum (top panel), high-field expansion (bottom panel)

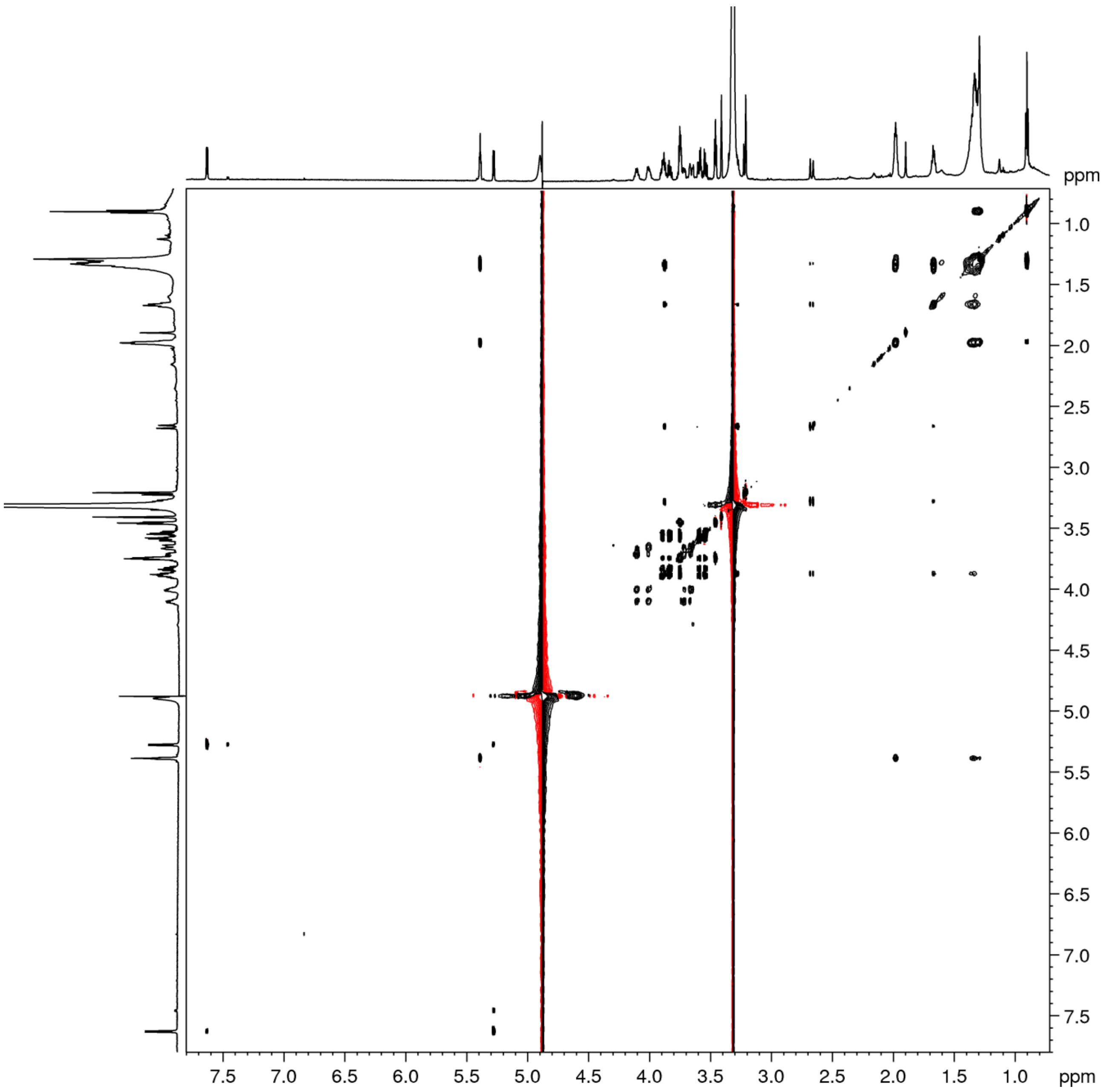

Figure S4. TOCSY spectrum of favilipid A (**1**) (700 MHz, CD_3OD).

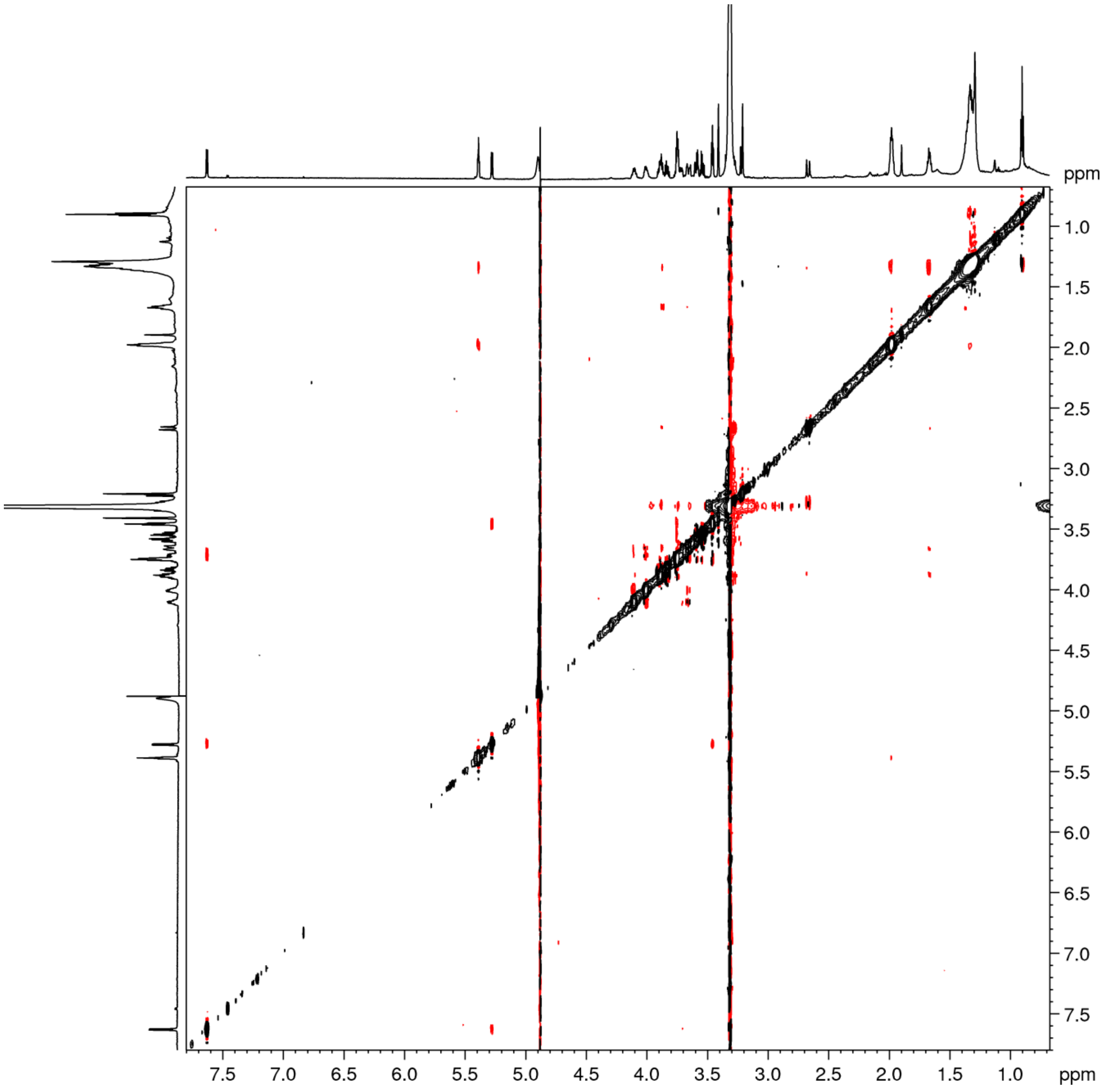

Figure S5. ROESY spectrum of favilipid A (**1**) (700 MHz, CD_3OD).

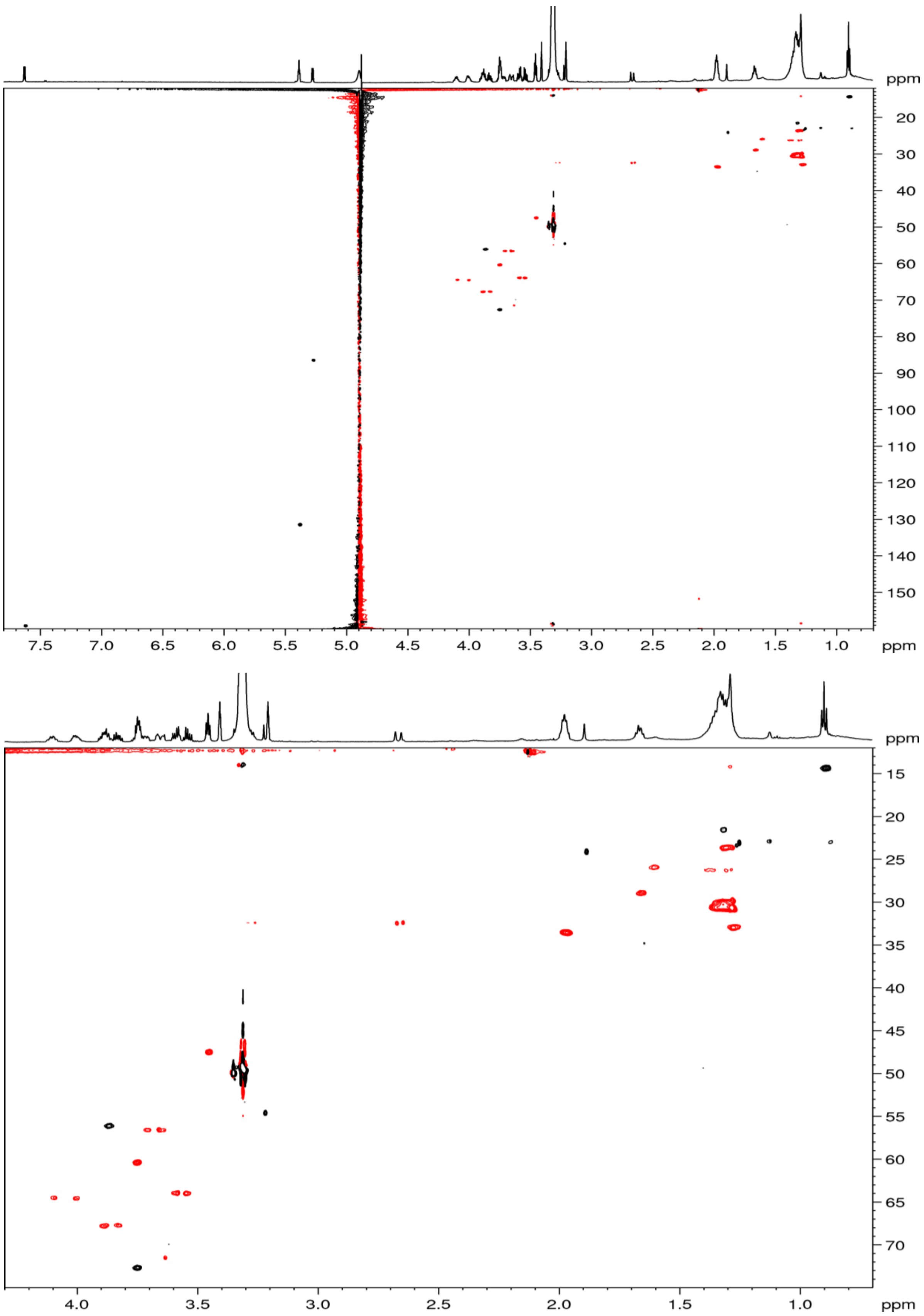

Figure S6. ^{13}C-HSQC spectrum of favilipid A (**1**) (700 MHz, CD_3OD): full spectrum (top panel), high-field expansion (bottom panel)

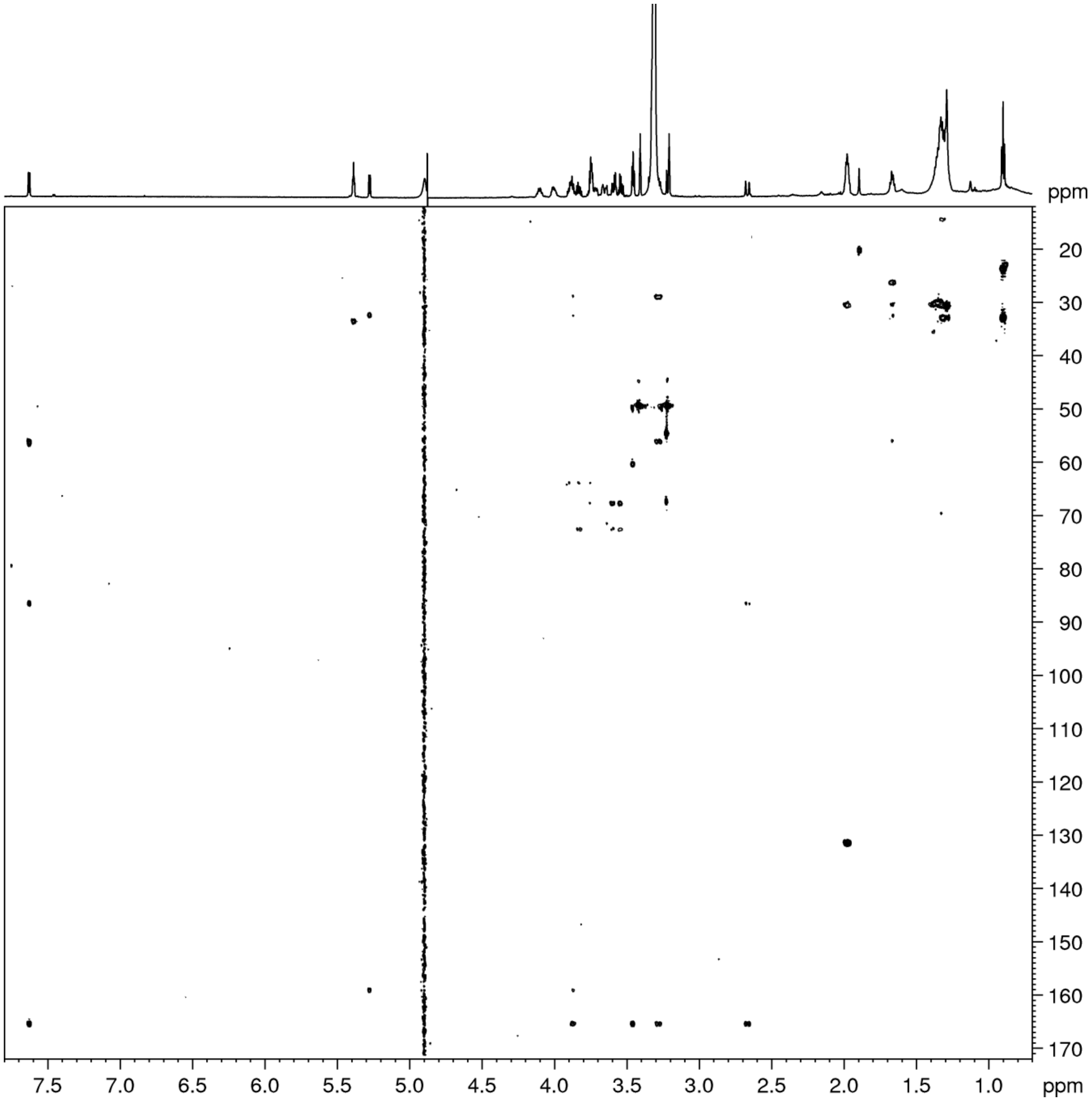

Figure S7. ^{13}C-HMBC spectrum of favilipid A (**1**) (700 MHz, CD_3OD).

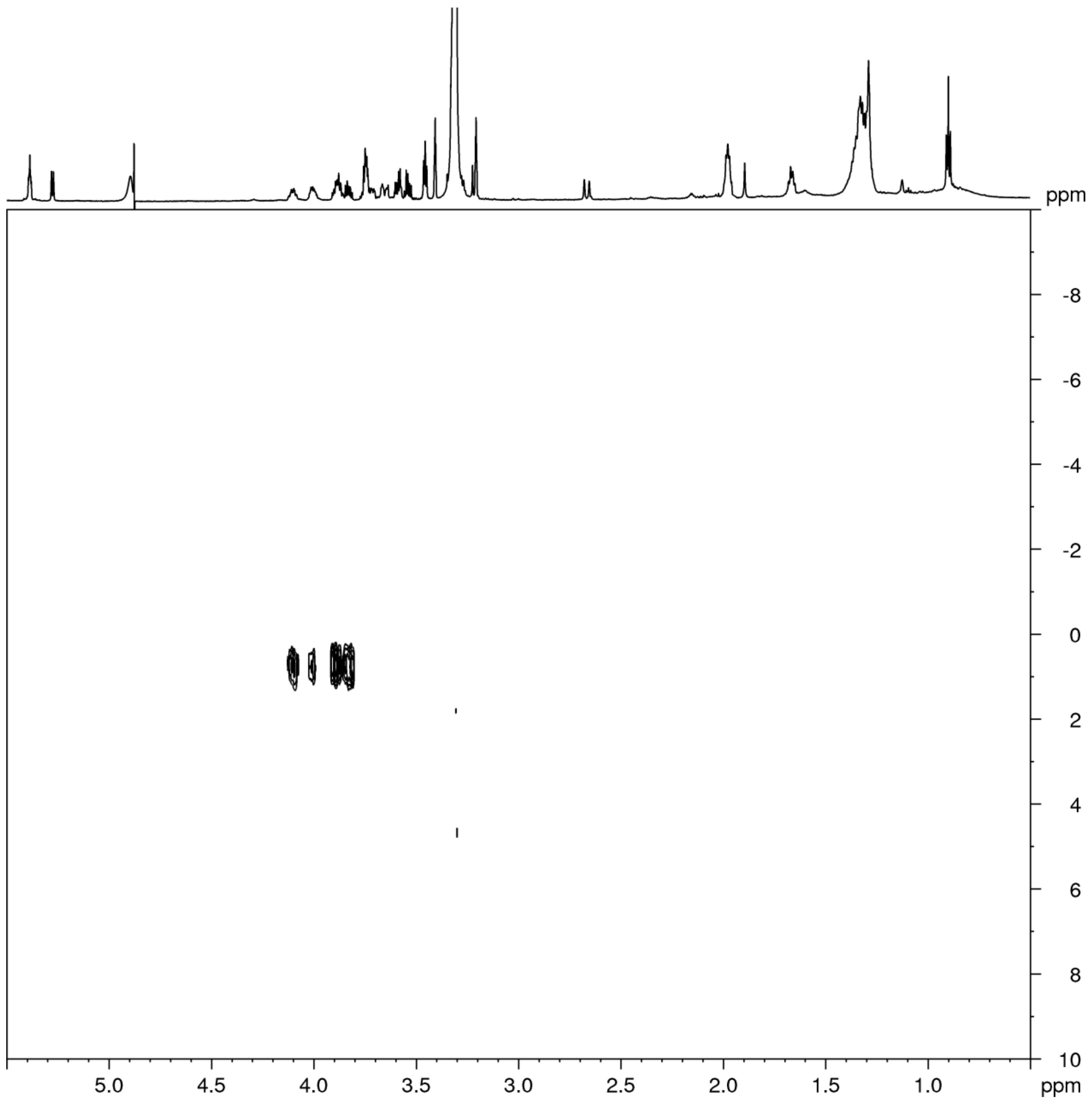

Figure S8. ^{31}P-HMBC spectrum of favilipid A (**1**) (700 MHz, CD_3OD).

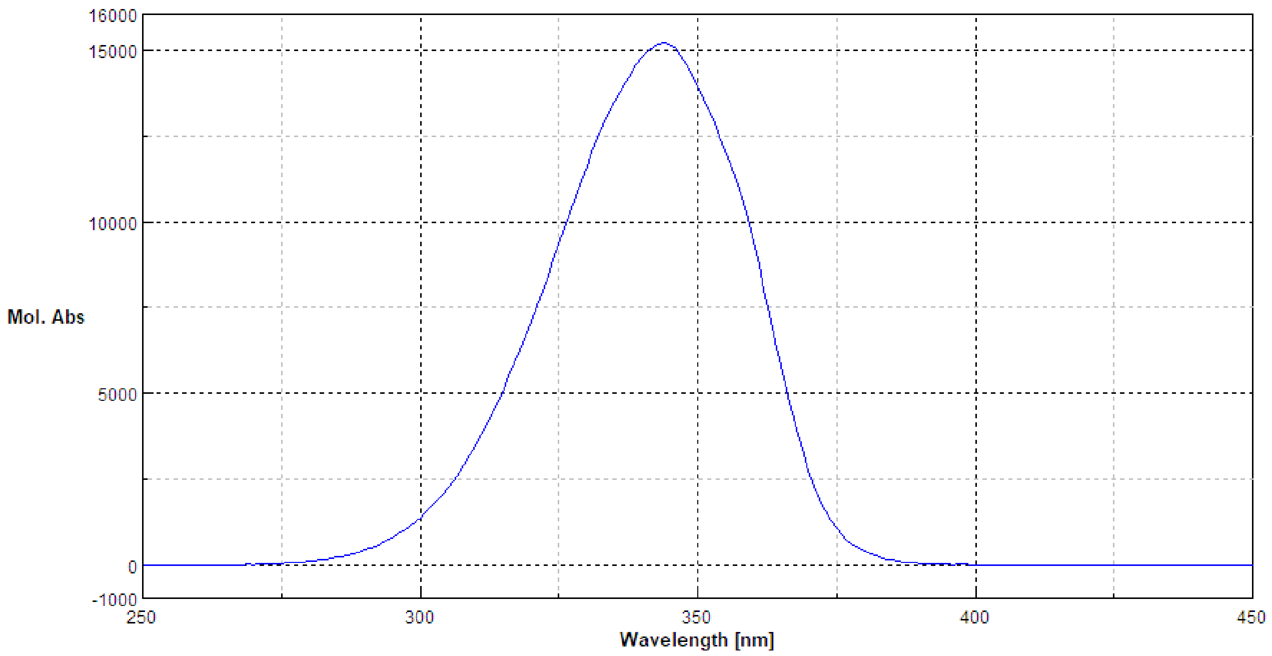

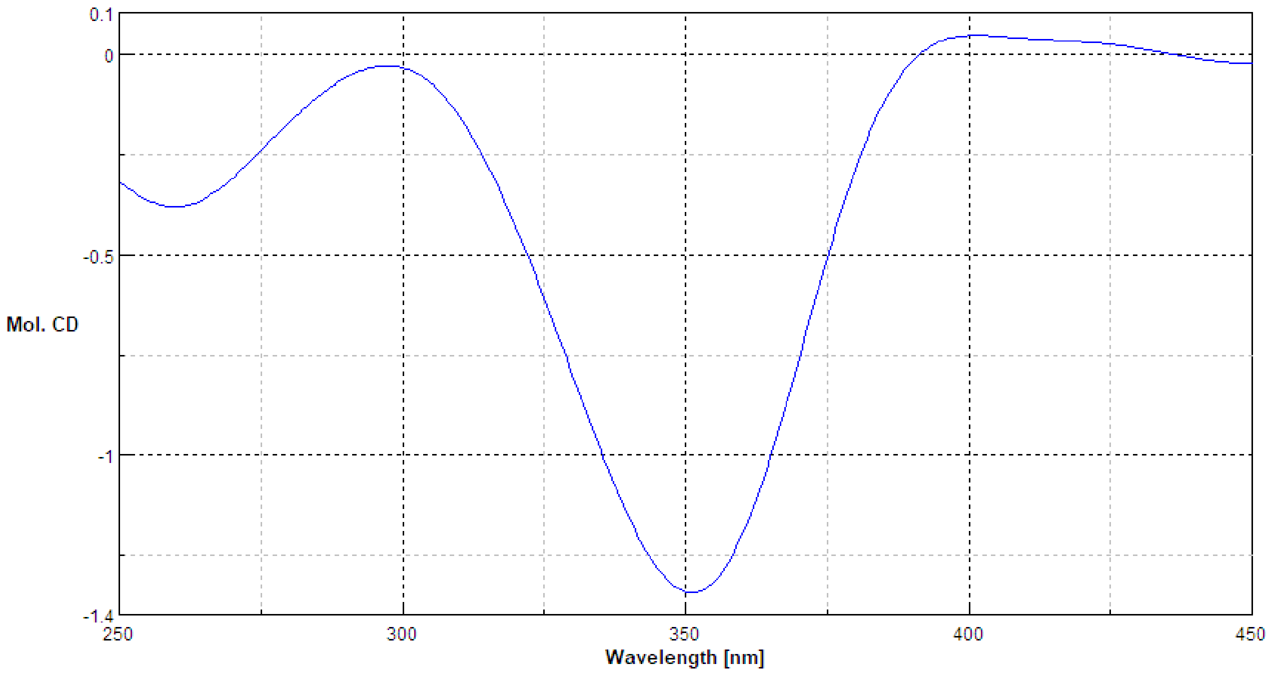

Figure S9. UV and ECD spectra of favilipid A (**1**) in MeOH.

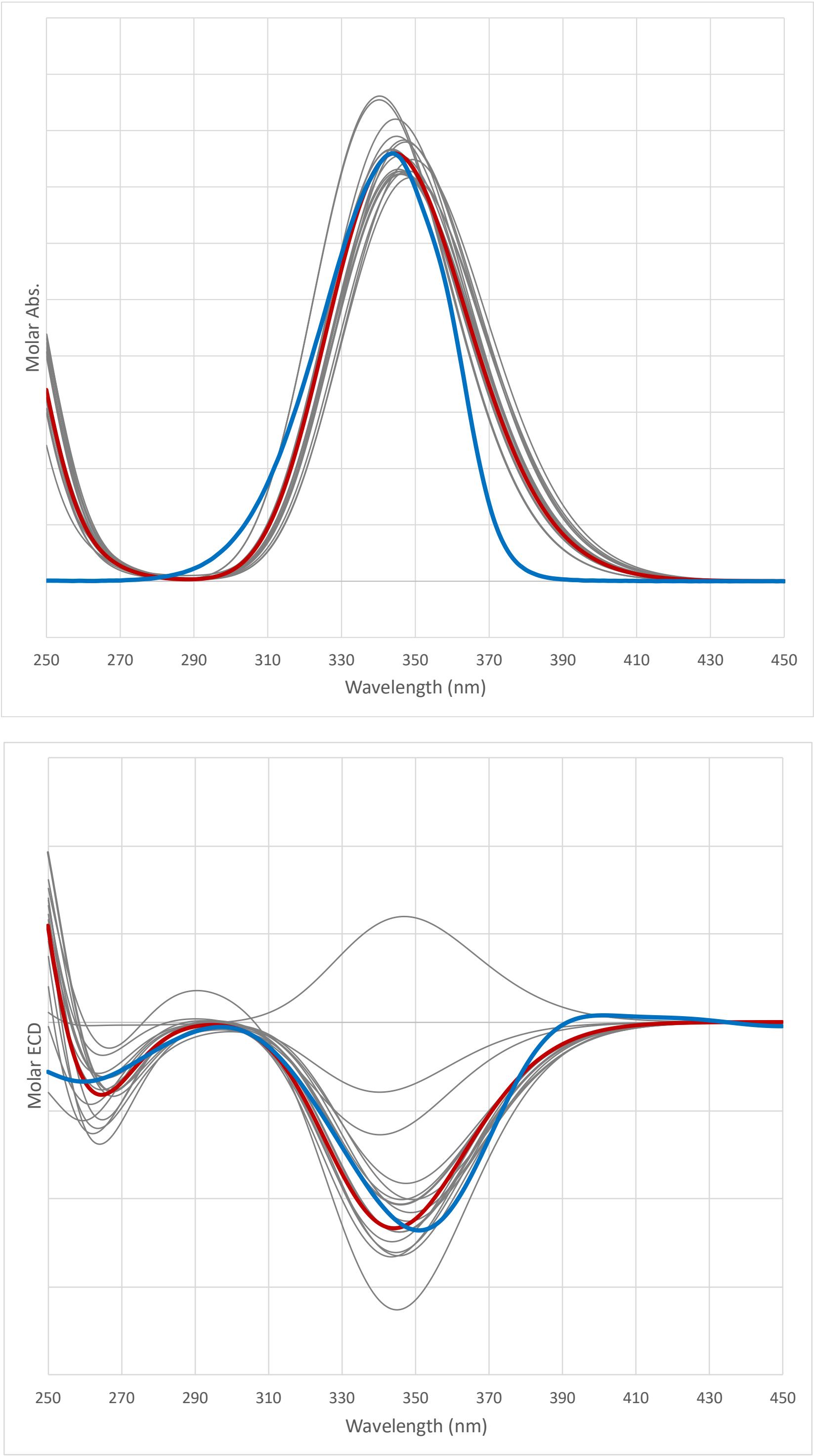

Figure S10. UV (top panel) and ECD (bottom panel) spectra of individual conformers and predicted average spectra of favilipid A (**1**) in MeOH.
Grey lines: spectra of individual conformers calculated at the B3LYP/6-311+G(2d,p)/SMD(MeOH)//B3LYP/6-31G(d,p)/SMD(MeOH) level of theory. The half-band width σ = 0.35 eV was used to generate the spectra. All the predicted spectra are shifted by +37 nm to match experimental and predicted UV maxima (see text for details). The only conformer showing positive ECD at 350 nm is conformer #11, whose population is 1.3%.
Red line: average UV and ECD spectra according to the Boltzmann statistics and B3LYP/6-31G+(d,p)/SMD(MeOH) energies.
Blue line: experimental UV and ECD spectra.

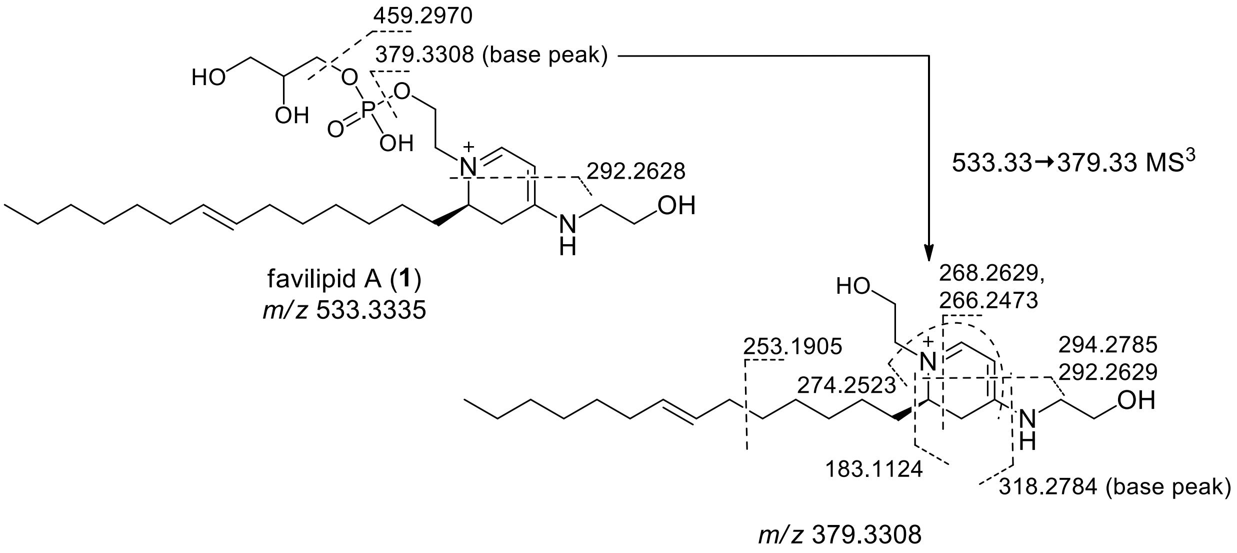

Figure S11. Interpretation of MS^2 and MS^3 spectra of favilipid A (**1**).

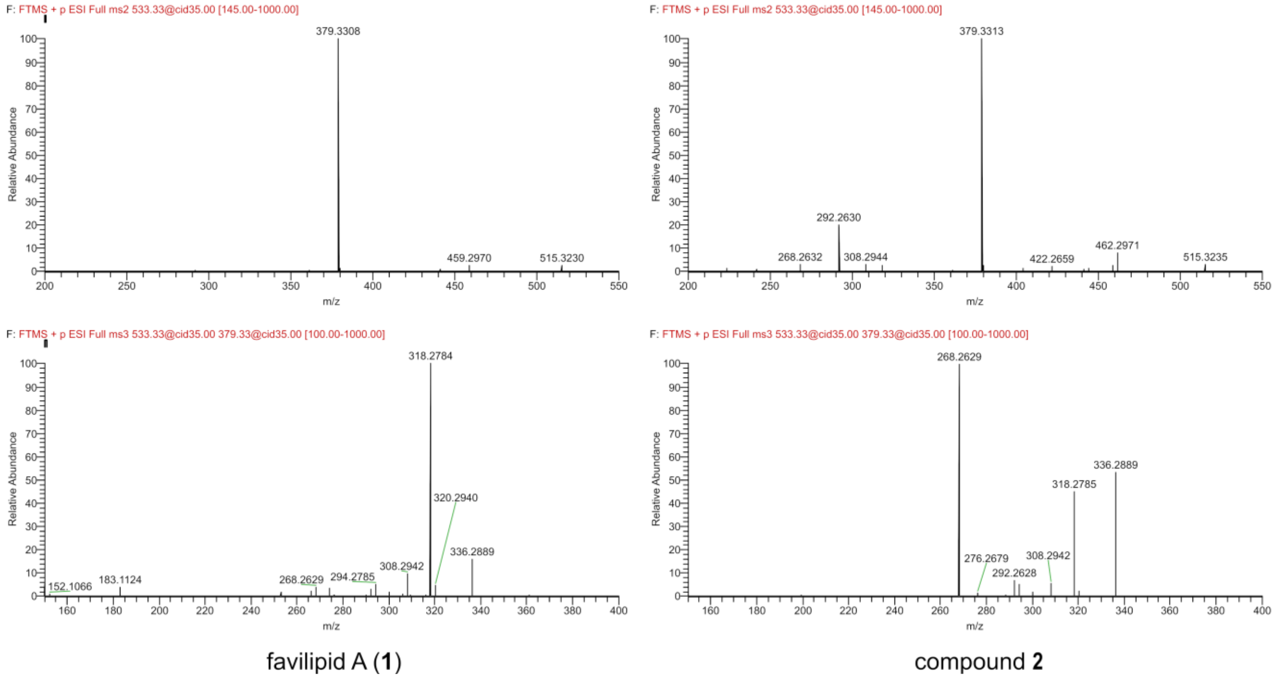

Figure S12. 533.33→MS^2 and 533.33→379.33→MS^3 spectra of favilipid A (**1**) and of its isomer **2**.

Table S1. Cartesian coordinates of the 16 low-energy conformers of the model compound **1m** of favilipid A used for prediction of the ECD spectrum.

favilipd model compound conf #01
Energy at B3LYP/6-31+G(d,p)/SMD(MeOH): -1183.7668160 Hartree

C	-1.30690	0.27700	-1.52780
C	-2.29580	1.16970	-1.10170
C	-2.79880	1.04880	0.19640
C	-2.16920	0.01790	1.09990
C	-1.72000	-1.23230	0.33240
C	-2.86800	-2.17390	-0.09050
C	-3.55430	-2.86550	1.09200
C	-4.37220	2.94700	0.00270
C	0.15250	-1.66790	-1.33080
C	1.44410	-1.43710	-0.55910
C	5.44960	-0.12950	0.72050
H	-0.78660	0.46130	-2.46380
H	-2.58670	1.97420	-1.76430
H	-1.29700	0.49250	1.56950
H	-2.85090	-0.26400	1.90600
H	-1.03020	-1.78770	0.97320
H	-5.11370	3.39190	0.66640
H	-2.44770	-2.93800	-0.75450
H	-3.60290	-1.61180	-0.67970
H	-4.31150	-3.56890	0.72900
H	-2.83240	-3.43240	1.69240
H	-3.63750	3.71020	-0.27490
H	-4.87260	2.58850	-0.90240
H	-4.05900	-2.15460	1.75490
H	-0.13870	-2.71660	-1.21250
H	0.31350	-1.47720	-2.39500
H	1.29040	-1.57330	0.51570
H	2.19330	-2.15800	-0.90510
H	6.20210	-0.82180	0.33750
H	5.22250	-0.37580	1.76250
H	5.83350	0.89430	0.65650
H	-3.99690	1.69180	1.67600
N	-0.95000	-0.81740	-0.86990
N	-3.73300	1.84170	0.70870
O	4.28100	-0.29400	-0.11010
O	1.90710	-0.09800	-0.82710
O	3.16490	2.03790	-0.38260
O	2.53650	0.49000	1.63180
P	2.96050	0.64630	0.18260

favilipd model compound conf #02
Energy at B3LYP/6-31+G(d,p)/SMD(MeOH): -1183.7654640 Hartree

C	1.35780	-0.06340	1.41000
C	2.20670	1.00740	1.11290
C	2.58020	1.22010	-0.21770
C	1.98860	0.31120	-1.26760
C	1.78290	-1.11360	-0.73720
C	3.11190	-1.88740	-0.61180
C	2.98400	-3.35100	-0.18110
C	3.91950	3.24520	0.25200
C	-0.00180	-2.00700	0.86080
C	-1.33150	-1.61420	0.23000
C	-5.26700	0.12130	-0.77190
H	0.89330	-0.12610	2.39010
H	2.47110	1.69540	1.90500
H	1.02340	0.74460	-1.56320
H	2.61920	0.28130	-2.16060
H	1.12520	-1.64480	-1.42990
H	4.52820	3.91340	-0.35710
H	3.77820	-1.35730	0.07960
H	3.58560	-1.84200	-1.60020
H	3.96800	-3.83250	-0.19960
H	2.59410	-3.44810	0.83780
H	3.12860	3.82580	0.73850
H	4.55070	2.78860	1.02090
H	2.32640	-3.91330	-0.85470
H	0.28420	-2.98920	0.47790
H	-0.10850	-2.07880	1.94670
H	-1.22670	-1.48530	-0.85160
H	-2.06260	-2.40660	0.42520
H	-6.04200	-0.63500	-0.63240

H	-5.01390	0.19350	-1.83450
H	-5.63500	1.08810	-0.41170
H	3.53060	2.30710	-1.61510
N	1.06830	-1.04670	0.56680
N	3.35170	2.22270	-0.62060
O	-4.12630	-0.31380	-0.00250
O	-1.78640	-0.38750	0.83510
O	-2.98810	1.82210	0.94950
O	-2.29220	0.87240	-1.38840
P	-2.78020	0.63540	0.02970

favilipd model compound conf #03
Energy at B3LYP/6-31+G(d,p)/SMD(MeOH): -1183.7646274 Hartree

C	1.12930	0.26360	1.45560
C	2.00890	1.28860	1.09300
C	2.62880	1.23460	-0.15820
C	2.23600	0.11400	-1.08910
C	1.90700	-1.18220	-0.33700
C	3.13520	-1.94820	0.20000
C	4.02960	-2.51930	-0.90500
C	3.87810	3.34970	0.12650
C	-0.01360	-1.87310	1.17920
C	-1.27640	-1.80810	0.33240
C	-4.57150	2.11960	0.02220
H	0.51450	0.37330	2.34450
H	2.12550	2.13130	1.76160
H	1.34850	0.45240	-1.64080
H	3.01820	-0.07370	-1.82830
H	1.36060	-1.83590	-1.02220
H	4.59890	3.89590	-0.48210
H	2.76760	-2.77190	0.82300
H	3.71800	-1.29030	0.85650
H	4.83310	-3.12030	-0.46540
H	3.45940	-3.16790	-1.58120
H	3.01680	3.99570	0.32700
H	4.34770	3.07710	1.07680
H	4.50020	-1.73460	-1.50680
H	0.42560	-2.86950	1.07210
H	-0.26300	-1.72080	2.23260
H	-1.03750	-1.90300	-0.73280
H	-1.93660	-2.63320	0.62290
H	-4.59290	2.97250	0.70310
H	-5.52870	1.59030	0.07090
H	-4.39280	2.47240	-0.99870
H	3.83670	2.04170	-1.54740
N	0.98800	-0.87450	0.79030
N	3.47310	2.15570	-0.60820
O	-3.49270	1.26990	0.46770
O	-1.92690	-0.54720	0.58360
O	-2.72710	0.19990	-1.77540
O	-4.34340	-1.09770	-0.20150
P	-3.19830	-0.11230	-0.36490

favilipd model compound conf #04
Energy at B3LYP/6-31+G(d,p)/SMD(MeOH): -1183.7645088 Hartree

C	-1.87210	1.17080	-1.32900
C	-3.08380	1.31040	-0.64460
C	-3.29660	0.55170	0.51010
C	-2.15440	-0.29600	1.01280
C	-1.30690	-0.86690	-0.13090
C	-1.96170	-2.03190	-0.90430
C	-2.12440	-3.30540	-0.06810
C	-5.56750	1.43250	0.94080
C	0.30170	0.17880	-1.81130
C	1.38390	1.05640	-1.19300
C	5.54780	0.80150	0.32960
H	-1.62660	1.86200	-2.13150
H	-3.79520	2.04690	-0.99440
H	-1.52760	0.34950	1.64320
H	-2.51820	-1.10590	1.64960
H	-0.36200	-1.21330	0.29270
H	-6.33200	1.23060	1.69100
H	-1.32770	-2.25360	-1.77060
H	-2.93370	-1.70980	-1.29770
H	-2.51270	-4.11770	-0.69220
H	-1.16300	-3.63280	0.34560
H	-5.30250	2.49450	0.97750
H	-5.96920	1.19950	-0.05040

H	-2.82260	-3.17120	0.76490
H	0.63230	-0.86140	-1.84560
H	0.13430	0.51720	-2.83900
H	2.27390	1.01540	-1.82850
H	1.04570	2.09550	-1.13430
H	6.05720	1.76160	0.22440
H	5.82570	0.14740	-0.50290
H	5.84000	0.33880	1.27850
H	-4.44330	0.02860	2.07380
N	-0.97370	0.22750	-1.08160
N	-4.40810	0.59680	1.23490
O	4.13290	1.08390	0.30790
O	1.69180	0.65030	0.15830
O	2.97410	-0.54560	1.96770
O	3.35490	-1.23630	-0.53010
P	3.07150	-0.16160	0.50460

favilipd model compound conf #05
Energy at B3LYP/6-31+G(d,p)/SMD(MeOH): -1183.7643324 Hartree

C	1.30210	-0.05690	1.30160
C	2.26150	0.92510	1.03740
C	2.79340	1.02230	-0.24950
C	2.22070	0.13200	-1.32350
C	1.70950	-1.22260	-0.80450
C	2.78570	-2.32270	-0.67680
C	3.93710	-2.07550	0.30290
C	4.29330	2.91630	0.27810
C	-0.13620	-1.94920	0.78380
C	-1.44620	-1.53520	0.12740
C	-5.55710	0.04690	-0.39530
H	0.77880	-0.04900	2.25350
H	2.52140	1.61830	1.82640
H	1.38650	0.68610	-1.77430
H	2.95350	-0.04170	-2.11690
H	0.97640	-1.58290	-1.53050
H	5.03690	3.47880	-0.28650
H	3.18690	-2.47290	-1.68730
H	2.27930	-3.25810	-0.40920
H	4.60090	-2.94740	0.31580
H	4.54320	-1.20790	0.02110
H	3.52650	3.60670	0.64540
H	4.78180	2.43870	1.13330
H	3.57780	-1.92290	1.32630
H	0.12390	-2.95340	0.43760
H	-0.25760	-1.98420	1.86960
H	-1.33140	-1.44110	-0.95690
H	-2.20300	-2.29860	0.34030
H	-6.28760	-0.68810	-0.05140
H	-5.47440	-0.00240	-1.48580
H	-5.88100	1.04850	-0.09270
H	4.00120	1.92520	-1.57870
N	0.97140	-1.03740	0.47270
N	3.71050	1.91410	-0.60770
O	-4.30280	-0.29890	0.22870
O	-1.85940	-0.27410	0.69020
O	-3.07600	1.92750	0.77040
O	-2.73300	0.74690	-1.54200
P	-2.98840	0.65470	-0.04810

favilipd model compound conf #06
Energy at B3LYP/6-31+G(d,p)/SMD(MeOH): -1183.7636565 Hartree

C	1.29970	0.67030	1.41450
C	2.28220	1.47570	0.83880
C	3.21570	0.89200	-0.02070
C	3.17260	-0.60380	-0.18740
C	1.75290	-1.17890	-0.09380
C	1.77340	-2.71570	-0.07250
C	2.44270	-3.33160	-1.30770
C	4.42700	2.99530	-0.49060
C	-0.13870	-1.24690	1.65780
C	-1.35330	-1.17920	0.74060
C	-5.50740	0.20420	-0.08530
H	0.65860	1.09290	2.18220
H	2.33140	2.51670	1.13000
H	3.61010	-0.88700	-1.14690
H	3.80250	-1.04070	0.60180
H	1.18010	-0.84350	-0.97190
H	2.28380	-3.05450	0.83830

H	0.74760	-3.09120	-0.02680
H	2.29980	-4.41740	-1.30160
H	2.00170	-2.94390	-2.23400
H	3.52040	-3.14340	-1.33940
H	3.54570	3.56040	-0.80940
H	4.66430	3.25880	0.54550
H	5.27050	3.26170	-1.12750
H	0.07660	-2.29300	1.88290
H	-0.36470	-0.74770	2.60260
H	-1.15940	-1.65560	-0.22440
H	-2.18370	-1.69930	1.23160
H	-6.18420	-0.11350	0.71050
H	-5.59700	-0.47930	-0.93570
H	-5.76770	1.22130	-0.39780
H	4.83300	1.03410	-1.20410
N	1.06050	-0.60080	1.09900
N	4.19220	1.56060	-0.62070
O	-4.17540	0.16080	0.46760
O	-1.69190	0.20770	0.53580
O	-2.86300	2.14070	-0.58130
O	-2.88270	-0.20720	-1.74290
P	-2.90900	0.62940	-0.47670

favilipd model compound conf #07
Energy at B3LYP/6-31+G(d,p)/SMD(MeOH): -1183.7635054 Hartree

C	1.92800	0.01090	1.61320
C	3.06340	0.68210	1.14990
C	3.21010	0.88480	-0.22660
C	2.08760	0.42970	-1.12740
C	1.41370	-0.84320	-0.60000
C	2.28790	-2.09710	-0.81130
C	1.64430	-3.42440	-0.40050
C	5.35400	2.10500	-0.04280
C	-0.18690	-1.17120	1.38090
C	-1.28180	-0.11410	1.46240
C	-5.35100	1.25810	0.36260
H	1.69930	0.01250	2.67640
H	3.75280	1.09730	1.87320
H	1.35640	1.24830	-1.17390
H	2.44760	0.25780	-2.14580
H	0.46710	-0.97410	-1.12730
H	6.05650	2.52020	-0.76560
H	3.24130	-1.97080	-0.28380
H	2.52360	-2.12680	-1.88250
H	2.30650	-4.25710	-0.66250
H	1.46450	-3.47930	0.67850
H	5.01160	2.90450	0.62280
H	5.86320	1.33980	0.55110
H	0.68920	-3.58470	-0.91450
H	-0.51520	-1.99990	0.75250
H	-0.00420	-1.55460	2.39030
H	-2.16560	-0.56310	1.92560
H	-0.95540	0.73320	2.07410
H	-5.69230	2.05290	1.02900
H	-5.75790	0.29910	0.69830
H	-5.69210	1.46920	-0.65670
H	4.22260	1.65130	-1.78300
N	1.07850	-0.65170	0.83830
N	4.23470	1.52410	-0.77720
O	-3.90950	1.25440	0.43380
O	-1.59080	0.40370	0.15030
O	-2.96480	0.73450	-1.93200
O	-3.59200	-1.20730	-0.29070
P	-3.07130	0.20070	-0.51710

favilipd model compound conf #08
Energy at B3LYP/6-31+G(d,p)/SMD(MeOH): -1183.7634115 Hartree

C	1.78020	-0.14540	1.75500
C	2.80790	0.68650	1.30050
C	2.92000	0.93050	-0.07170
C	1.85450	0.36210	-0.97510
C	1.33160	-0.99190	-0.47950
C	2.28980	-2.18080	-0.70650
C	2.50560	-2.51660	-2.18560
C	4.89680	2.40230	0.12810
C	-0.08710	-1.69360	1.52130
C	-1.39510	-0.92730	1.64790
C	-4.14180	1.19720	-2.13610

H	1.57940	-0.21630	2.82120
H	3.44430	1.17060	2.02950
H	1.02870	1.08640	-0.99280
H	2.21870	0.26920	-2.00090
H	0.39130	-1.19660	-0.99530
H	5.53280	2.92390	-0.58720
H	1.86030	-3.05360	-0.20120
H	3.25140	-1.97490	-0.22040
H	3.12250	-3.41690	-2.28080
H	1.55210	-2.71020	-2.69170
H	4.45990	3.13290	0.81710
H	5.50690	1.69490	0.69850
H	3.01660	-1.71170	-2.72460
H	-0.22160	-2.56800	0.87980
H	0.19810	-2.04380	2.51780
H	-2.13390	-1.57550	2.13410
H	-1.25210	-0.02840	2.25810
H	-3.86560	1.18530	-3.19210
H	-4.23120	2.23390	-1.79510
H	-5.09620	0.67800	-2.00260
H	3.82160	1.84330	-1.61780
N	1.01120	-0.88780	0.96950
N	3.85560	1.70000	-0.61480
O	-3.08370	0.51360	-1.43070
O	-1.85480	-0.55800	0.33310
O	-4.44060	-0.40880	0.58650
O	-2.97470	1.72460	0.86990
P	-3.19980	0.37880	0.20030

favilipd model compound conf #09
Energy at B3LYP/6-31+G(d,p)/SMD(MeOH): -1183.7632852 Hartree

C	1.19600	0.04410	1.34260
C	1.93380	1.20080	1.07290
C	2.43980	1.39170	-0.21650
C	2.10330	0.35960	-1.26550
C	2.00610	-1.05030	-0.66780
C	3.39430	-1.63800	-0.33790
C	3.39630	-3.08280	0.16860
C	3.44820	3.59530	0.27310
C	0.16810	-2.08800	0.77600
C	-1.13160	-1.89470	0.00600
C	-4.37890	2.05940	0.46550
H	0.62900	-0.02780	2.26660
H	2.01180	1.95770	1.84230
H	1.14000	0.64910	-1.70690
H	2.84420	0.36350	-2.07040
H	1.50780	-1.69780	-1.39370
H	4.03940	4.30710	-0.30300
H	3.89850	-0.99150	0.39070
H	3.97800	-1.58020	-1.26510
H	4.42710	-3.43190	0.29540
H	2.89850	-3.18060	1.13930
H	2.53790	4.09080	0.62700
H	4.03210	3.26330	1.13730
H	2.90180	-3.75840	-0.53950
H	0.61130	-3.03790	0.46980
H	-0.03760	-2.13380	1.84890
H	-0.93540	-1.79580	-1.06740
H	-1.77130	-2.76930	0.17180
H	-4.34480	2.80570	1.26150
H	-5.33770	1.53200	0.50110
H	-4.26240	2.55520	-0.50380
H	3.41400	2.52290	-1.56170
N	1.13470	-1.00960	0.53820
N	3.12320	2.46620	-0.59210
O	-3.28460	1.15100	0.71220
O	-1.78270	-0.71000	0.50290
O	-2.62340	0.40250	-1.69080
O	-4.24260	-1.05870	-0.27130
P	-3.06490	-0.09990	-0.32600

favilipd model compound conf #10
Energy at B3LYP/6-31+G(d,p)/SMD(MeOH): -1183.7632841 Hartree

C	-0.93440	0.42410	-1.05210
C	-1.92610	1.38520	-0.82770
C	-2.82760	1.19160	0.22190
C	-2.61510	-0.00310	1.11910
C	-2.05350	-1.21000	0.35640

C	-3.07650	-1.94520	-0.53600
C	-4.18730	-2.64500	0.25370
C	-4.08370	3.28350	-0.17920
C	0.20740	-1.70140	-0.69530
C	1.22110	-1.69080	0.44480
C	4.60390	2.17580	0.19060
H	-0.12830	0.63600	-1.74880
H	-1.91980	2.28560	-1.42780
H	-1.90160	0.30270	1.89640
H	-3.54080	-0.28090	1.62880
H	-1.65910	-1.91680	1.09150
H	-4.96240	3.74340	0.27300
H	-2.52990	-2.69350	-1.12140
H	-3.51020	-1.23770	-1.25310
H	-4.82680	-3.21910	-0.42560
H	-3.77150	-3.34330	0.99030
H	-3.23850	3.97560	-0.10190
H	-4.28760	3.08800	-1.23670
H	-4.83010	-1.93560	0.78580
H	-0.19990	-2.71340	-0.78870
H	0.69660	-1.44110	-1.63650
H	0.73660	-1.90810	1.40100
H	1.97040	-2.46740	0.25830
H	4.41620	3.19140	0.54410
H	4.82680	2.19850	-0.88120
H	5.45100	1.75000	0.73800
H	-4.37750	1.82200	1.33550
N	-0.90370	-0.76960	-0.47640
N	-3.80400	2.03870	0.52840
O	3.39770	1.42550	0.44810
O	1.83630	-0.39300	0.58310
O	4.35940	-0.95850	0.85090
O	3.41750	-0.31410	-1.48640
P	3.36550	-0.15910	0.02480

favilipd model compound conf #11
Energy at B3LYP/6-31+G(d,p)/SMD(MeOH): -1183.7632499 Hartree

C	-1.51970	-1.51660	-0.28250
C	-2.68140	-1.33980	0.47560
C	-3.51870	-0.26070	0.17750
C	-3.14350	0.61480	-0.99370
C	-1.62140	0.75330	-1.13000
C	-0.98810	1.64960	-0.04100
C	-1.38150	3.12520	-0.17120
C	-5.21680	-0.82260	1.88430
C	0.10910	-0.93600	-1.99780
C	1.45600	-0.36400	-1.58020
C	4.13510	1.78930	1.23210
H	-0.97410	-2.45340	-0.21580
H	-2.94720	-2.09700	1.20150
H	-3.55800	0.14400	-1.89570
H	-3.60410	1.60170	-0.90860
H	-1.39970	1.17920	-2.11230
H	-6.14350	-0.35590	2.21850
H	0.09920	1.56590	-0.11200
H	-1.26510	1.27350	0.95100
H	-0.84190	3.72300	0.57130
H	-1.12710	3.51750	-1.16350
H	-5.43200	-1.84100	1.54370
H	-4.51690	-0.86830	2.72450
H	-2.45170	3.29020	-0.00720
H	0.17950	-2.02500	-2.05110
H	-0.12440	-0.56380	-3.00090
H	2.19870	-0.68300	-2.31930
H	1.44380	0.72950	-1.54860
H	3.75500	2.79520	1.42150
H	4.92900	1.83440	0.47980
H	4.53030	1.37000	2.16360
H	-5.21520	0.77290	0.48050
N	-1.02010	-0.60930	-1.11280
N	-4.66550	-0.01650	0.80000
O	3.01780	1.01290	0.75160
O	1.79260	-0.89060	-0.28180
O	3.28900	-1.39950	1.67680
O	4.37120	-0.72070	-0.60980
P	3.24840	-0.58010	0.40240

favilipd model compound conf #12

Energy at B3LYP/6-31+G(d,p)/SMD(MeOH): -1183.7631985 Hartree

C	1.32020	0.34420	1.36870
C	2.23400	1.31270	0.95310
C	3.13180	1.00460	-0.07350
C	3.15000	-0.41410	-0.57260
C	1.76210	-1.06680	-0.55270
C	1.83320	-2.55140	-0.94960
C	2.69230	-3.45790	-0.06020
C	4.19850	3.23420	-0.11680
C	-0.05840	-1.62620	1.17230
C	-1.30770	-1.33670	0.34940
C	-5.49260	0.08920	-0.09610
H	0.70620	0.54530	2.24140
H	2.25830	2.25710	1.48120
H	3.54410	-0.46360	-1.59140
H	3.84360	-0.96750	0.07440
H	1.13110	-0.55360	-1.29350
H	0.81710	-2.95240	-1.01860
H	2.22510	-2.56850	-1.97360
H	2.60930	-4.49500	-0.40350
H	3.75260	-3.18760	-0.09660
H	2.37650	-3.43090	0.98880
H	3.27260	3.80050	-0.25770
H	4.48350	3.27280	0.93980
H	4.98820	3.68850	-0.71510
H	0.17490	-2.68890	1.08700
H	-0.25390	-1.41550	2.22630
H	-1.13890	-1.51470	-0.71640
H	-2.10880	-1.99910	0.69660
H	-6.15760	-0.49380	0.54430
H	-5.54880	-0.28940	-1.12170
H	-5.79680	1.14120	-0.07190
H	4.65000	1.51570	-1.28470
N	1.11230	-0.82860	0.77110
N	4.02860	1.85330	-0.55830
O	-4.16220	-0.07390	0.43780
O	-1.68330	0.03970	0.55890
O	-2.92300	2.18160	0.08400
O	-2.84690	0.32030	-1.75540
P	-2.91130	0.71300	-0.29060

favilipd model compound conf #13

Energy at B3LYP/6-31+G(d,p)/SMD(MeOH): -1183.7631374 Hartree

C	1.55640	0.08520	-1.52040
C	2.54430	-0.83300	-1.15410
C	2.82400	-1.02040	0.20130
C	1.95170	-0.31640	1.20990
C	1.39940	1.03460	0.72480
C	2.34560	2.23730	0.92890
C	3.69060	2.21230	0.19610
C	4.62740	-2.67080	-0.17520
C	-0.13080	1.80070	-1.16280
C	-1.48740	1.11320	-1.19820
C	-4.08180	-2.36440	-0.45670
H	1.24190	0.14140	-2.55960
H	3.03380	-1.40440	-1.93170
H	1.11420	-0.99360	1.42450
H	2.48950	-0.16580	2.15070
H	0.49870	1.23760	1.30710
H	5.31810	-3.20460	0.47770
H	2.51660	2.30640	2.01100
H	1.79960	3.14670	0.64960
H	4.24150	3.13550	0.40910
H	4.32090	1.37500	0.51420
H	4.04510	-3.39840	-0.75050
H	5.20120	-2.04640	-0.86720
H	3.56390	2.15080	-0.89020
H	-0.16960	2.67580	-0.51080
H	0.10400	2.14220	-2.17560
H	-2.22330	1.81120	-1.61190
H	-1.44880	0.21880	-1.83000
H	-3.64780	-3.26450	-0.89680
H	-4.83220	-1.94990	-1.13720
H	-4.54980	-2.61890	0.50040
H	3.85590	-1.93610	1.66330
N	0.95620	0.92790	-0.69130
N	3.75660	-1.84810	0.65810
O	-2.99410	-1.43530	-0.26840

O	-1.86130	0.74130	0.14440
O	-3.38720	-0.13430	1.94500
O	-4.42740	0.71810	-0.30360
P	-3.30260	0.01960	0.43910

favilipd model compound conf #14
Energy at B3LYP/6-31+G(d,p)/SMD(MeOH): -1183.7624681 Hartree

C	-1.86310	-0.95700	1.15080
C	-3.16150	-1.32330	0.78730
C	-3.75630	-0.69760	-0.31290
C	-2.91620	0.26800	-1.11160
C	-1.92580	1.04290	-0.23250
C	-2.56170	2.16080	0.62170
C	-3.11010	3.32520	-0.20960
C	-5.88700	-1.94010	-0.14290
C	0.17400	0.37740	1.03560
C	1.15730	-0.20090	0.01770
C	6.25350	0.38610	0.17860
H	-1.32760	-1.54120	1.89460
H	-3.64290	-2.12910	1.32550
H	-2.36300	-0.32500	-1.85280
H	-3.54570	0.96630	-1.66790
H	-1.17160	1.48820	-0.88670
H	-6.83000	-1.91210	-0.68870
H	-1.79130	2.54160	1.30210
H	-3.35530	1.73590	1.24830
H	-3.47280	4.12030	0.45090
H	-2.33190	3.75440	-0.85230
H	-5.46910	-2.95050	-0.20320
H	-6.07400	-1.69390	0.90700
H	-3.94750	3.02410	-0.84820
H	0.29950	1.46210	1.09580
H	0.36610	-0.04700	2.02450
H	1.09660	-1.29530	0.01580
H	0.94200	0.17140	-0.98980
H	6.76850	1.20520	0.68390
H	6.50960	0.39780	-0.88610
H	6.55660	-0.56670	0.62390
H	-5.30600	-0.47860	-1.57280
N	-1.21130	0.08760	0.65560
N	-4.98190	-0.96650	-0.74520
O	4.84100	0.61420	0.37020
O	2.46940	0.22890	0.42580
O	4.02260	-1.84630	0.20220
O	3.70340	-0.23120	-1.81440
P	3.78210	-0.43670	-0.31060

favilipd model compound conf #15
Energy at B3LYP/6-31+G(d,p)/SMD(MeOH): -1183.7623296 Hartree

C	-1.84030	0.43870	1.51330
C	-2.77620	-0.55930	1.22870
C	-2.80180	-1.11150	-0.05640
C	-1.76440	-0.63930	-1.04580
C	-1.40960	0.83920	-0.84180
C	-2.52100	1.78240	-1.34790
C	-2.19610	3.27710	-1.27730
C	-4.61850	-2.71550	0.43540
C	-0.02480	1.97470	1.00000
C	1.26290	1.24380	1.35100
C	4.11930	-1.68010	-1.75860
H	-1.67360	0.73920	2.54490
H	-3.39320	-0.93730	2.03330
H	-0.87100	-1.26170	-0.89980
H	-2.10410	-0.79410	-2.07380
H	-0.48780	1.04850	-1.38800
H	-5.15940	-3.45620	-0.15360
H	-3.44970	1.58030	-0.80020
H	-2.70560	1.50250	-2.39260
H	-3.00610	3.85700	-1.73370
H	-2.08710	3.62790	-0.24550
H	-4.13680	-3.21520	1.28250
H	-5.32830	-1.97410	0.81530
H	-1.27230	3.51400	-1.81820
H	0.15520	2.67710	0.18530
H	-0.34450	2.54290	1.87940
H	1.99710	1.97960	1.70030
H	1.08500	0.51210	2.14710
H	3.83240	-1.98350	-2.76720

H	4.29390	-2.57140	-1.14670
H	5.03080	-1.07560	-1.80410
H	-3.54030	-2.43660	-1.37400
N	-1.11490	1.07110	0.59930
N	-3.62620	-2.08300	-0.42780
O	3.01760	-0.90610	-1.23750
O	1.75540	0.57360	0.17410
O	4.34600	0.59970	0.41220
O	2.97120	-1.41970	1.31360
P	3.13700	-0.31160	0.28710

favilipd model compound conf #16

Energy at B3LYP/6-31+G(d,p)/SMD(MeOH): -1183.7621729 Hartree

C	1.15900	-0.00440	1.25970
C	2.00970	1.07020	0.98500
C	2.61180	1.15290	-0.27150
C	2.22270	0.13620	-1.31490
C	1.83590	-1.23650	-0.73780
C	3.02010	-2.19300	-0.47910
C	4.07260	-1.75540	0.54420
C	3.84230	3.24080	0.21710
C	-0.00520	-2.08900	0.79140
C	-1.31590	-1.87540	0.04690
C	-4.42420	2.19340	0.45710
H	0.57820	-0.00270	2.17770
H	2.13320	1.83710	1.73830
H	1.36430	0.55810	-1.85460
H	3.02510	0.00280	-2.04660
H	1.19580	-1.72150	-1.47900
H	4.54500	3.85980	-0.34080
H	3.49800	-2.35370	-1.45410
H	2.60880	-3.16450	-0.17940
H	4.82700	-2.54270	0.65320
H	4.59560	-0.84280	0.23890
H	2.97640	3.84920	0.49900
H	4.33210	2.87600	1.12540
H	3.63460	-1.58340	1.53330
H	0.39240	-3.07060	0.51990
H	-0.18380	-2.08100	1.86980
H	-1.14690	-1.82960	-1.03480
H	-1.98550	-2.71480	0.26620
H	-4.41490	2.90460	1.28520
H	-5.41070	1.72230	0.39350
H	-4.20160	2.71820	-0.47750
H	3.79550	2.11220	-1.58320
N	1.00180	-1.06640	0.48150
N	3.43990	2.12620	-0.63400
O	-3.40480	1.21430	0.75000
O	-1.90460	-0.64480	0.51000
O	-2.76400	0.49300	-1.66530
O	-4.36790	-0.98790	-0.24870
P	-3.19280	-0.02580	-0.30260
